# Identifying and Quantifying Borate Environments in Borosilicate Glasses: ^11^B NMR-Peak Assignments Assisted by Double-Quantum Experiments

**DOI:** 10.1021/acs.jpcb.4c06721

**Published:** 2024-12-10

**Authors:** Baltzar Stevensson, Peng Lv, Mattias Edén

**Affiliations:** † Physical Chemistry Division, Department of Materials and Environmental Chemistry, Arrhenius Laboratory, Stockholm University, Stockholm SE-106 91, Sweden; ‡ MOE Frontiers Science Center for Rare Isotopes, Lanzhou University, Lanzhou 730000, PR China

## Abstract

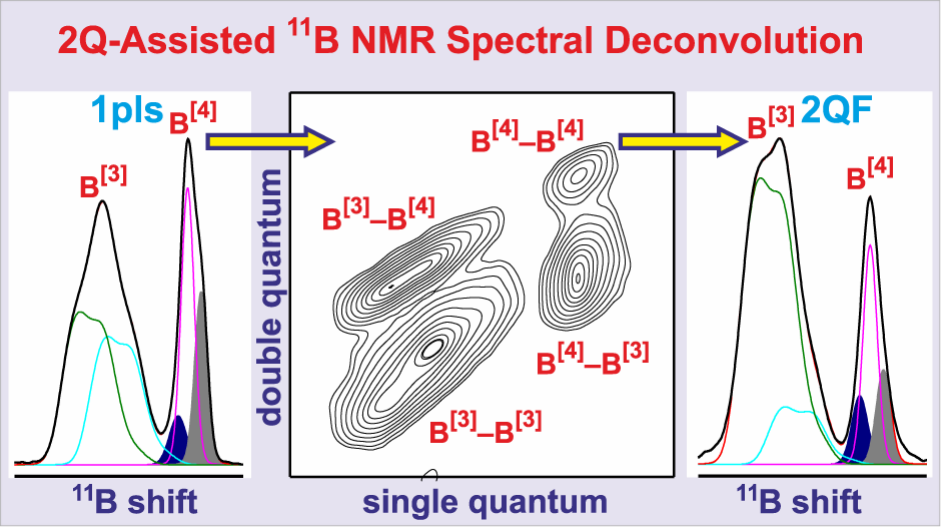

We discuss the prospects for accurate ^11^B magic-angle-spinning (MAS) nuclear magnetic resonance (NMR) spectral deconvolutions for reaching beyond the readily extracted borate speciations offered by the integrated resonances of the coexisting B^[3]^ and B^[4]^ species of the respective BO_3_ and BO_4_ network groups in borosilicate (BS) glasses. We critically review hitherto proposed ^11^B^[3]^ and ^11^B^[4]^ NMR-peak assignments relating to their neighboring Si, B^[3]^ and B^[4]^ species, as quantified by MAS NMR spectral deconvolution. Guidance to these resonance assignments was offered from double-quantum–single-quantum (2Q–1Q) ^11^B MAS NMR experiments that inform about the B^[*p*]^–O–B^[*q*]^ network linkages. The NMR spectral deconvolutions from two BS glass series with low nonbridging oxygen (NBO) contents and fixed molar ratios *n*_Si_/*n*_B_ = {1.0, 2.0} but variable network-modifying cations of alkali metals and Mg^2+^ revealed a dominance of B^[4]^–O–Si linkages, yet with a significant dependence on the BO_3_ population of the glass, which was rationalized by the different propensities for B^[4]^–O–{Si, B^[3]^, B^[4]^} linkage formation. For BS glasses with comparable B and Si contents, we recommend three-peak deconvolutions of the ^11^B^[4]^ spectral region, whose ^11^B^[4]^(*m*Si) sites differ in their (average) numbers of *m* B^[4]^–O–Si and 4 – *m* B^[4]^–O–B^[*p*]^ bonds, where B^[*p*]^ may assume B^[3]^*or* B^[4]^. We also discuss the structural origin of the two rather arbitrarily classified “ring” and “non-ring” B^[3]^ entities, where 2Q–1Q ^11^B NMR suggests the former to primarily constitute BO_3_ groups that coexist with BO_4_ moieties in (superstructural) ring units largely devoid of bonds to Si, whereas the “non-ring” B^[3]^ sites involve linkages to all of B^[3]^, B^[4]^, and Si, with B^[3]^–O–Si linkages prevailing. The limitations of ^11^B NMR spectral deconvolutions are discussed, including the remaining challenges in analyzing NBO-rich BS glasses.

## 1 Introduction

Borosilicate (BS) glasses constitute networks of SiO_4_, BO_3_, and BO_4_ polyhedra that are interlinked by *bridging oxygen* (BO) atoms between the *network-forming* (*F*) species *F* = {B^[3]^, B^[4]^, Si ≡ Si^[4]^}, where the superscript denotes the coordination number with respect to O. The B^[3]^ and B^[4]^ populations of a B-bearing glass depend strongly on the precise glass composition,^1−4^ in particular on the amounts of electropositive *M*^*z*+^ cations, whose incorporation into a silicate/phosphate glass breaks up its network by converting BO atoms into *nonbridging* O (NBO; O^–^) anions, but for a BS glass also altering its borate speciation,^1−4^ and thereby the physical glass properties.^5−11^ Hence, there is a long-standing interest in characterizing not only the B^[3]^/B^[4]^ populations but also their B^[*p*]^–O–*F* bonding partners. For a clear-cut discrimination between the structural *M*^*z*+^ and *F* entities, we employ the old “network modifier” term when referring to the *M*^+^/*M*^2+^ cations.

The two most widely applied routine techniques for probing the local B environments in BS-based glasses, encompassing those with additional network formers (such as Al and P), are Raman^11−17^ and ^11^B nuclear magnetic resonance (NMR) spectroscopy,^6,10,17−43^ the latter nowadays normally performed under magic-angle-spinning (MAS) conditions.^2−4^ Both methods are complementary and have inherent (dis)advantages. The Raman vibrations are sensitive to network-group aggregates, referred to as “superstructural units”^1,44,45^ (Section 3.2), whereas the ^11^B central-transition (CT) MAS NMR spectral region readily permits discrimination and accurate quantification of the two B^[3]^/B^[4]^ coordinations.^2−4^ However, both Raman and ^11^B NMR spectra manifest insufficient spectral resolution and assignment ambiguities. Raman spectral resolution is compromised for all but the simplest *M*_2_O–B_2_O_3_ glasses,^13,16,45^ which complicates unambiguous spectral interpretations, which is also reflected in Raman band reassignments over time.^13−16,45^

Likewise, reaching beyond {B^[3]^, B^[4]^} speciation quantifications of BS glasses is nontrivial by routine ^11^B MAS NMR experiments, owing to the structural disorder that disperses the ^11^B^[3]^ and ^11^B^[4]^ chemical shifts and smears out the spectral responses, which, besides distributions of interpolyhedral bond angles and B^[*p*]^–O distances, stem both from variable numbers of B^[*p*]^–O–{B^[3]^, B^[4]^, Si} linkages and the BO/NBO distribution at the BO_3_ groups.^2−4^ Following pioneering multinuclear MAS NMR studies on the network-group intermixing,^17,21−23,25−27,46−48^ it is nowadays standard practice to attempt extracting more detailed information about the local ^11^B^[3]^ and ^11^B^[4]^ environments from a BS glass by deconvoluting its ^11^B CT NMR spectral region.^25−34,37−39,49^ For the B^[4]^ ensemble, such analyses aim at quantifying the relative populations of B^[4]^(*m*Si) ≡ ^11^B^[4]^(OSi)_*m*_(OB)_4–*m*_ motifs with *m* Si and 4 – *m* B neighbors, whose resonances overlap heavily across the narrow ^11^B^[4]^ CT MAS NMR region. Precise ^11^B^[3]^ resonance assignments are even more challenging, where two resonances are traditionally attributed to “*ring*” (R) and “*non-ring*” (NR) ^11^B^[3]^ sites in BS-based glasses,^25−34,37−39,49,50^ yet without any widespread consensus of their precise structural meaning because the R/NR concept originates from the much simpler case of vitreous B_2_O_3_,^51−54^ whose BO_3_-built structure presents no complexities from Si, B^[4]^, and NBO species.

Given the ambiguities of ^11^B MAS NMR spectral deconvolutions for gaining more detailed structural information about B^[*p*]^–O–*F* motifs, an attractive alternative is to exploit the magnetic ^11^B–^29^Si dipolar interaction, which is mediated directly through space and scales as the inverse cube of the ^11^B–^29^Si distance.^3,55^ Advanced NMR experimentation relying on such *hetero*nuclear dipolar interactions offers interconnectivity information on BS-based glass networks,^30,33,40,56−60^ and may also assist ^11^B^[4]^(*m*Si) NMR-peak assignments.^30,33^ However, probing of the B/Si intermixing in BS glasses via ^11^B–^29^Si dipolar interactions remains sparse, mainly because high-quality NMR data enabling quantitative analyses are normally precluded by the low natural abundance of the NMR-active ^29^Si isotope (4.7%), necessitating glass preparation from very expensive ^29^SiO_2_.^33,40,58^ Another option for gaining B/Si interconnectivity information is ^17^O triple-quantum MAS^61^ (3QMAS),^24−27,35,36,62−66^ which, besides requiring costly isotopic ^17^O enrichment, may not unambiguously discriminate between the two *p* = {3, 4} coordinations of B^[*p*]^–O–Si and B^[*p*]^–O–B^[*q*]^ linkages (although such B^[*p*]^ assignments have been claimed from multipeak fitting^25−27,63^).

Following our recent work on the identification and quantification of borate-group linkages in BS-based glasses,^41,67,68^ by utilizing the *homo*nuclear ^11^B–^11^B interactions in double-quantum–single-quantum (2Q–1Q) correlation ^11^B NMR experiments,^69−72^ we here investigate to what extent they may assist resonance assignments of routinely acquired “single-pulse” (Bloch-decay) ^11^B MAS NMR spectra. The 1D analog of a 2Q–1Q 2D NMR acquisition resulting from solely employing the *t*_1_ = 0 data point is termed a *double-quantum filtration* (2QF) NMR experiment,^71,72^ yielding the projection along the horizontal 2D NMR spectral dimension. Although not revealing the detailed B^[3]^–O–B^[3]^, B^[3]^–O–B^[4]^, and B^[4]^–O–B^[4]^ linkages from a B-bearing glass, its recording is at least 10^1^ times shorter than arranging the 2D NMR spectrum.^71,72^ The 2QF NMR spectrum only comprises resonances from ^11^B^[*p*]^ sites featuring *at least one* B^[*p*]^–O–B^[*q*]^ linkage, whose intensities emphasize concurrently with the *number* of B^[*p*]^(OB) bonds. In contrast, all ^11^B NMR signals from (for instance) **B**^[4]^(OSi)_4_, **B**^[3]^(OSi)_3_, and **B**^[3]^(OSi)_2_ NBO sites are absent, despite that they appear in the standard Bloch-decay MAS NMR spectrum. Notwithstanding that ^11^B/^29^Si-based NMR has a decisive advantage of *directly* informing about B^[*p*]^–O–Si interconnectivities, 2QF ^11^B NMR experimentation is readily applied to *any* glass prepared from inexpensive oxide precursors (thanks to the high ^11^B natural abundance of 80%), while the receptivity of the ^11^B nuclide is superior relative to both ^29^Si and ^17^O.^2−4^

Herein, ^11^B MAS NMR spectra recorded from nine BS glass specimens (Section 2) in the presence and absence of 2QF were deconvoluted. They constitute a subset of a large ensemble of ternary *M*_2_O–B_2_O_3_–SiO_2_ and quaternary *M*_(2)_O–Na_2_O–B_2_O_3_–SiO_2_ glasses with variable B, Si, and NBO contents,^10^ for which several physical properties were reported and their composition–property relationships discussed,^10^ along with their borate-group interconnectivities.^68^ Besides two Mg-bearing specimens, all glasses herein are alkali-metal based, furnishing two series with fixed molar ratios of *n*_Si_/*n*_B_ = 1.0 and *n*_Si_/*n*_B_ = 2.0, while exhibiting (very) low NBO contents, which, besides simplifying ^11^B NMR spectral deconvolutions, conform to the mainstream BS-glass composition range targeted in previous ^11^B NMR spectral deconvolutions.^25−33^

## 2 Materials and Methods

### 2.1 Present Borosilicate Glasses

Table 1 presents the nominal compositions of the 9 BS glasses targeted herein, which are expressed both as their oxide equivalents and the sets of atomic fractions of each element *E* in the *M*–(Na)–B–Si–O glass,
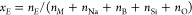
1where *n*_*E*_ is the corresponding stoichiometric amount. We employ the {*K*, *R*} parameters
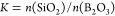
2
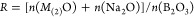
3generalized from the definitions introduced by Bray and co-workers for *R*Na_2_O–B_2_O_3_–*K*SiO_2_ glasses.^19,20^ The glass specimens exhibit a fixed *R* = 0.75 value along with either *K* = 2.0 or *K* = 4.0. Each ternary 0.75Na_2_O–B_2_O_3_–*K*SiO_2_ and 0.75K_2_O–B_2_O_3_–*K*SiO_2_ glass is denoted by Na*K* and K*K*, respectively, whereas each quaternary 0.75[0.5*M*_(2)_O–0.5Na_2_O]–B_2_O_3_–*K*SiO_2_ member is labeled *M*Na*K* with *M*^*z*+^ = {Rb^+^, Li, Mg^2+^}.

**Table 1:** Borosilicate Glass Compositions and Borate Speciations^a^

	Oxide Equivalents (mol %)				Atomic Fractions						
Glass	*M*_(2)_O	Na_2_O	B_2_O_3_	SiO_2_	*x*_*M*_	*x*_Na_	*x*_B_	*x*_Si_	*x*_O_		*x*_NBO_
K2.0	20.0		26.7	53.3	0.114		0.151	0.151	0.584	0.628	0.031
RbNa2.0	10.0	10.0	26.7	53.3	0.057	0.057	0.151	0.151	0.584	0.603	0.038
Na2.0		20.0	26.7	53.3		0.114	0.151	0.151	0.584	0.604	0.038
LiNa2.0	10.0	10.0	26.7	53.3	0.057	0.057	0.151	0.151	0.584	0.563	0.048
MgNa2.0	10.0	10.0	26.7	53.3	0.029	0.058	0.155	0.155	0.603	0.361	0.100
											
K4.0	13.0		17.4	69.6	0.078		0.104	0.208	0.610	0.681	0.012
RbNa4.0	6.5	6.5	17.4	69.6	0.039	0.039	0.104	0.208	0.610	0.651	0.017
Na4.0		13.0	17.4	69.6		0.078	0.104	0.208	0.610	0.618	0.022
MgNa4.0	6.5	6.5	17.4	69.6	0.020	0.040	0.106	0.212	0.622	0.313	0.074

a Nominal compositions of ternary 0.75*M*_2_O–B_2_O_3_–*K*SiO_2_ glasses—denoted by *MK*—and quaternary 0.75[0.5*M*_(2)_O–0.5Na_2_O]–B_2_O_3_–*K*SiO_2_ glasses—denoted by *M*Na*K*, along with the corresponding atomic fractions {*x*_*M*_, *x*_Na_, *x*_B_, *x*_Si_, *x*_O_} given by Equation 1.  (uncertainty ±0.01) and *x*_NBO_ (uncertainty ±0.01) represent the ^11^B NMR-derived fractional population of B^[4]^ coordinations and NBO species, respectively. The  values were obtained from the integrated ^11^B^[*p*]^ NMR intensities of the Bloch-decay NMR spectra and were corrected for the signal intensity of the satellite-transition centerband (Section 2.3), whereas the NBO fraction was calculated from the expression .

The borate speciation of a BS glass depends strongly on the *M*^*z*+^*cation field strength* (CFS),^73^
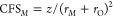
4where *r*_O_ = 1.36 Å^73^ and *r*_*M*_ is the cation radius for the (as-assumed) 6-fold coordination (*M*^[6]^) with charge *z*.^74^ All figures/tables herein list the specimens within each *K* = {2.0, 4.0} series from top to bottom according to increasing *averageM*^*z*+^/Na^+^ CFS:  = (CFS_*M*_ + CFS_Na_)/2, whose values are listed in Table S1.

Table 1 also presents the ^11^B MAS NMR-derived *fractional population* of B^[4]^ coordinations () out of the entire borate speciation of the glass; the remaining constitutes B^[3]^ species, whose respective population () is obtained from the normalization condition
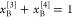
5All glass networks but those of the two Mg-bearing glasses are dominated by BO_4_ groups. From each  value, the fractional population of NBO anions (O^[1]^ coordinations) out of all NBO and BO (O^[2]^) species was extracted via ,^10,68^ where *x*_BO_ + *x*_NBO_ = 1. The NBO fractions are listed in Table 1.

### 2.2 Glass Preparation

All BS glass preparation and characterization procedures were identical to those reported previously by Lv et al.,^10,38,68^ to which we refer for details. The samples were prepared in batches of 5–8 g by melt-quenching from analytical grade (purity 99.9%+) precursors of SiO_2_, H_3_BO_3_, and anhydrous carbonates of the metal cations, except for Mg^2+^ which was introduced from MgO. After preheating at 950 °C for 24 h to remove potential OH/H_2_O contaminations of the SiO_2_ powder, the precursors were mixed thoroughly and subsequently decarbonated in a Pt crucible at 950 °C for 2 h, before heating up to the final temperature in the 1125–1275 °C range, where precise temperatures are given in ref (10). The melt was kept for 20–30 min and then quenched by immersing the crucible bottom in cold water. All glass specimens were free of crystalline impurities. From the NMR-analyzed B contents and negligible mass losses during heating, we concluded that all nominal stoichiometries listed in Table 1 are very close to the “physical” glass compositions,^10,68^ as confirmed by elemental analyses of all *K* = 4.0 glasses.^38^

### 2.3 Solid-State NMR Experiments

All ^11^B (spin-3/2) NMR experiments were performed with a Bruker Avance-III spectrometer at a magnetic field (*B*_0_) of 14.1 T (−192.5 MHz ^11^B Larmor frequency) using 3.2 mm zirconia rotors undergoing MAS at 24.00 kHz. Neat BF_3_·OEt_2_ was used for ^11^B shift referencing and for determining ^11^B nutation frequencies (ν_B_).

The {, } values of each glass were estimated from the integrated signal intensities of ^11^B MAS NMR spectra recorded by strong/short radio frequency (rf) pulses (0.33 μs; 13° flip angle; ν_B_ = 105 kHz), as reported and discussed previously by Lv et al.^10,68^ The only difference herein was the use of a larger zero filling of the time-domain data to improve the as-acquired data of ref (68), yielding a spectral resolution of 0.038 ppm. ^11^B NMR probehead “background” signals were eliminated by subtracting the NMR spectrum observed from the empty rotor under otherwise identical experimental conditions (such artifacts are removed identically by the 2QF process; *vide infra*). The as-integrated ^11^B CT NMR signal intensities were corrected for the satellite-transition (ST) centerband peak that overlaps with the main CT ^11^B^[4]^ signal by using standard procedures.^75^

The 2Q–1Q ^11^B NMR experiments utilized the rf-pulse protocol of Figure 2d of Edén^71^ with one completed 90°-pulse-sandwiched *S*R2_2_^1^ dipolar recoupling sequence,^70,76,77^ for excitation of two-spin CT 2Q coherences (2QC), along with equal 2QC excitation/reconversion intervals of τ_exc_ = 4τ_*r*_ = 167 μs, where  is the rotor period and ν_*r*_ is the spinning speed. The ^11^B–^11^B dipolar recoupling pulses operated at  kHz, while the CT-selective 90° (16 μs) and 180° (32 μs) pulses employed  kHz. The *t*_1_-evolution stage of each 2Q–1Q 2D NMR spectrum was preceded by a Hahn-echo of duration 2τ_*r*_ for ensuring rotor-synchronized 2QC excitation and reconversion stages,^70^ with the CT-selective 180° pulse of the echo segment cycled in 8 steps to eliminate all undesirable *single*-spin ST 2QC^69^ (ref (78) provides the shortest rf-phase cycle enabling that task by a standard nested phase cycling^79^). 1536–3456 NMR-signal transients were accumulated with relaxation delays of 1.5 s. All 2Q–1Q correlation ^11^B NMR spectra presented herein are reproduced from raw data published by Lv et al.^68^

### 2.4 ^11^B MAS NMR Spectral Fitting

The ^11^B CT MAS NMR region observed in directly excited spectra and upon 2QF (i.e., the horizontal projection of the 2Q–1Q NMR spectrum) was deconvoluted into five ^11^B^[*p*]^ NMR peaks for each glass: three components reflecting ^11^B^[4]^(*m*Si) ≡ ^11^B^[4]^(OSi)_*m*_(OB)_4–*m*_ environments with *m* = {2, 3, 4}, along with two “B^[3]^(R)” and “B^[3]^(NR)” contributions from trigonal ^11^B sites in “ring” or “non-ring” constellations, respectively. The spectral deconvolutions utilized software developed in our laboratory to represent the ^11^B^[3]^ and ^11^B^[4]^ NMR peak shapes by numerically exact simulations^80^ and efficient powder averaging.^81^ All ^11^B^[*p*]^ resonances were fitted with unconstrained populations, i.e., integrated intensities. The deconvolution of each unique NMR spectrum started from an initial fitting with unconstrained chemical shifts and quadrupolar parameters to locate reasonable ranges for subsequent parameter constraints.

The resulting best-fit parameters are collected in Table S2. Besides the two {(NR)} populations, the iterative fitting of each ^11^B^[3]^(R) and ^11^B^[3]^(NR) resonance involved four parameters: {}. They encompass the *quadrupolar coupling constant*, , the asymmetry parameter of the electric field gradient (efg) tensor, , and a Gaussian chemical-shift *distribution* with full width at half-maximum (fwhm) width of  and centered at the *isotropic chemical shift*.^4^ The chemical-shift distribution of all ^11^B^[3]^ and ^11^B^[4]^ sites was confined to 2.4 ± 0.6 ppm.  was restricted within 13.5–15.0 ppm, whereas  and the accompanying asymmetry parameters were unconstrained. Although the resulting best-fit  values conformed to 0.35 ± 0.1, reliable and physically meaningful asymmetry parameters (distributions) are not expected from these spectral deconvolutions.^4^ Consequently, we only give relevance to the *quadrupolar products* () of each {^11^B^[3]^(R), ^11^B^[3]^(NR), ^11^B^[4]^(*m*Si)} environment, defined as the root-mean-square (rms) value over the quadrupolar products of all such *N*^11^B_*j*_^[p]^ sites in the glass:^4^
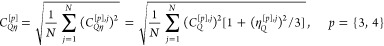
6

Each of the three ^11^B^[4]^(*m*Si) NMR-peak components with *m* = {2, 3, 4} was modeled by the four parameters {, , , }, where the efg tensor-parameters assumed a Czjzek distribution.^82,83^ The chemical shifts were constrained to ±0.25 ppm. Initial numerical fitting of the ^11^B^[4]^ NMR signal region without shift distributions  resulted in unreasonably large values . Besides implying that the set  governs all peak widths, the very small quadrupolar products could not be extracted by numerical fitting of the present ^11^B NMR spectra recorded at 14.1 T. Hence, based on previous rough assessments of  MHz,^67^ the values were (rather arbitrarily) fixed according to  = {0.24, 0.31, 037} MHz, along with expectations of a minor increase in quadrupolar products for decreasing symmetry in the second coordination sphere of B^[4]^.^31^ These findings also consolidate the most common approach of representing each ^11^B^[4]^(*m*Si) NMR peak by more readily implemented Gaussian (or Gaussian/Lorentzian) curves,^25,26,29,30,37,49,50,84^ which we recommend also for future deconvolutions.

High-quality spectral deconvolutions resulted for all single-pulse ^11^B MAS NMR spectra by representing the net ^11^B^[4]^ resonance by three peak components, which gave consistently better fits than the more common use of only two signals from the ^11^B^[4]^(3Si) and ^11^B^[4]^(4Si) sites (Sections 3.1 and 5.2). Although the resonance intensities of these NMR spectra quantitatively reflect the underlying site populations, these numerical multiparameter fits should not be taken too literally because the parameter uncertainties—estimated as the standard error of the rms deviation between the best fit and experiment—only reflect their as-assumed/selected number of peak components.

The numerical fitting of the 2Q–1Q NMR spectral projections (2QF NMR spectra), however, left some unaccounted signal regions at the outermost high/low ppm ranges of the respective ^11^B^[3]^/^11^B^[4]^ resonance flanks, which are essentially artifacts of the 2D NMR spectra. There are also more fundamental obstacles compromising the determination of nuclear-site populations by 2QF NMR experiments in the networks of amorphous inorganic materials, notably so for quadrupolar nuclides,^71,72^ such as ^11^B. The fractional-population set of {B^[3]^–O–B^[3]^, B^[3]^–O–B^[4]^, B^[4]^–O–B^[4]^} linkages in a BS glass may be estimated reasonably accurately via their corresponding integrated 2Q–1Q ^11^B NMR spectral intensities by applying a correction procedure^67,68^ that accounts for an overrepresented ^11^B^[3]^ spectral intensity upon 2QF application and mainly stemming from the higher  values relative to .^76,85^ Such corrections, however, are not possible for the 2QF NMR spectra. We may consequently only make qualitative comparisons among the *relative* integrated 2QF NMR intensities (“apparent site populations”) *within* each group of trigonal {B^[3]^(R), B^[3]^(NR)} and tetrahedral {B^[4]^(2Si), B^[4]^(3Si), B^[4]^(4Si)} sites, whose members manifest very similar quadrupolar products around ∼2.7 MHz and <0.5 MHz, respectively (Table S2).

## 3 Previously Proposed ^11^B MAS NMR Assignment Options

### 3.1 B^[4]^ Environments

A hitherto incompletely settled issue concerns the unambiguous identification of the populations of the set of coexisting ^11^B^[4]^(*m*Si) environments with *m* Si and 4 – *m* B^[*p*]^ neighbors and their accompanying isotropic chemical shifts, {(*m*Si)}, along with accurate experimental determination of their populations. The very narrow ^11^B^[4]^-resonance span (Section 4.1) complicates even the decision of *how many* distinct B^[4]^(*m*Si) moieties coexist, while the relative numbers of bonds to B/Si atoms in the second coordination sphere of ^11^B^[4]^ affect its chemical shift.^2−4^ However, except for a few reports explicitly stating the assumed sole presence of B^[4]^–O–{B^[3]^, Si} linkages (i.e., absence of B^[4]^–O–B^[4]^),^25−27,49,50,86^ the literature is very vague concerning the precise meaning and coordination of “B” of the typically employed “B^[4]^(*m*Si, [4 – *m*]B)” notation,^28,32,36,37^ which we herein abbreviate by B^[4]^(*m*Si) and discuss in relation to hitherto largely unaccounted B^[4]^–O–B^[4]^ bonds.^67,68^ Likewise, for NBO-rich BS glasses, potential ^11^B^[4]^ shift effects from NBO bonds are currently implicitly/explicitly ignored, although such bonds are predicted from atomistic MD simulations^41,67,68,87−89^ (Section 5.3). Fortunately, B^[4]^–NBO bonding is irrelevant for our present NBO-poor BS glasses, as well as for most of those in the literature invoking ^11^B MAS NMR spectral deconvolutions.^25−33^

Two or three distinct-*m* B^[4]^(*m*Si) environments are normally introduced for deconvoluting the ^11^B^[4]^ NMR spectral region, but the degree of ^11^B^[4]^*deshielding* (increased chemical shift) per ^11^**B**^[4]^–O–Si → ^11^**B**^[4]^–O–B^[3]^ substitution remains ill-defined, where 0.5–1.0 ppm,^22,33^ ≈1.5 ppm,^37,38^ and 1.7–2.0 ppm^25−27,31,32^ have been used. The consensus is that ^11^B^[4]^(4Si) environments exhibit shifts within −1.8 ± 0.3 ppm for BS glasses with low NBO contents (*x*_NBO_ ≲ 0.1),^25−27,30−33,37,38^ whereas the precise number and chemical shifts of the other ^11^B^[4]^(*m*Si) environments are less defined. ^11^B NMR analyses of nearly NBO-free and Si-rich BS glasses (such as all *K* = 4.0 members herein) often assumed *two* peak components from B^[4]^(4Si) and B^[4]^(3Si) motifs.^25,28−33^ Moreover, within that assumption of solely *m* = {3, 4} groups being present, Angeli et al.^31^ demonstrated a simple and efficient approach for their discrimination by utilizing a Hahn spin–echo, where the ^11^B^[4]^(4Si) signal manifests a slower *T*_2_ relaxation-stemming NMR-signal decay than ^11^B^[4]^(3Si).^31^

To support the ^11^B^[4]^(*m*Si) NMR-peak assignments in spectra from Si-*rich* BS glasses, additional information about the degree of B^[4]^–O–Si bonding, gauged from dipolar-based double-resonance ^11^B/^29^Si NMR experimentation suggested that either B^[4]^(3Si) groups,^30^ or B^[4]^(2Si) along with B^[4]^(1Si) environments,^33^ coexisted with the B^[4]^(4Si) moieties. For the very silica-rich (83 mol %; *K* = 7.2) Pyrex glass, Tricot^30^ deduced from such experiments in conjunction with ^11^B MAS NMR spectral fitting, that {δ_B_^[4]^(3Si), δ_B_^[4]^(4Si)} ≈ {−0.25, −2.0} ppm. In contrast, from double rotation^90,91^^11^B–^11^B correlation NMR applied to Pyrex glass, Howes et al.^34^ assigned a resonance at δ_B_^[4]^ ≈ −1.4 ppm as merely stemming from *both* B^[4]^(3Si) and B^[4]^(4Si) moieties, along with another δ_B_^[4]^ ≈ 0.5 ppm signal that was attributed to ^11^BO_4_ groups in superstructural units without B^[*p*]^–O–Si linkages.^34^ Hence, ^11^B^[4]^ NMR-peak assignments are far from settled, *even* for the most widely examined Si-rich/NBO-poor BS-based glass composition domain.

Du and Stebbins^26,27^ introduced *three*-peak deconvolutions of the ^11^B^[*p*]^-resonance region with {, , } ≈ {1.5, −0.2, −2.2} ppm, i.e., with a 1.7–2.0 ppm shift separation between neighboring *m* ± 1 values. Krishnamurthy and Kroeker^37^ employed a slightly different shift triplet, fixed at {1.3, −0.1, −1.7} (±0.2) ppm, for analyzing aluminoborosilicate glasses, which is very close to that recently employed by some of us^38^ and amounts to a  ppm deshielding for each ^11^**B**^[4]^–O–Si → ^11^**B**^[4]^–O–B replacement. That is also well aligned with earlier studies concluding that (3Si) ≈ 0.0 ± 0.5 ppm.^25,28,30−32^

### 3.2 B^[3]^ Environments

**Figure 1:**
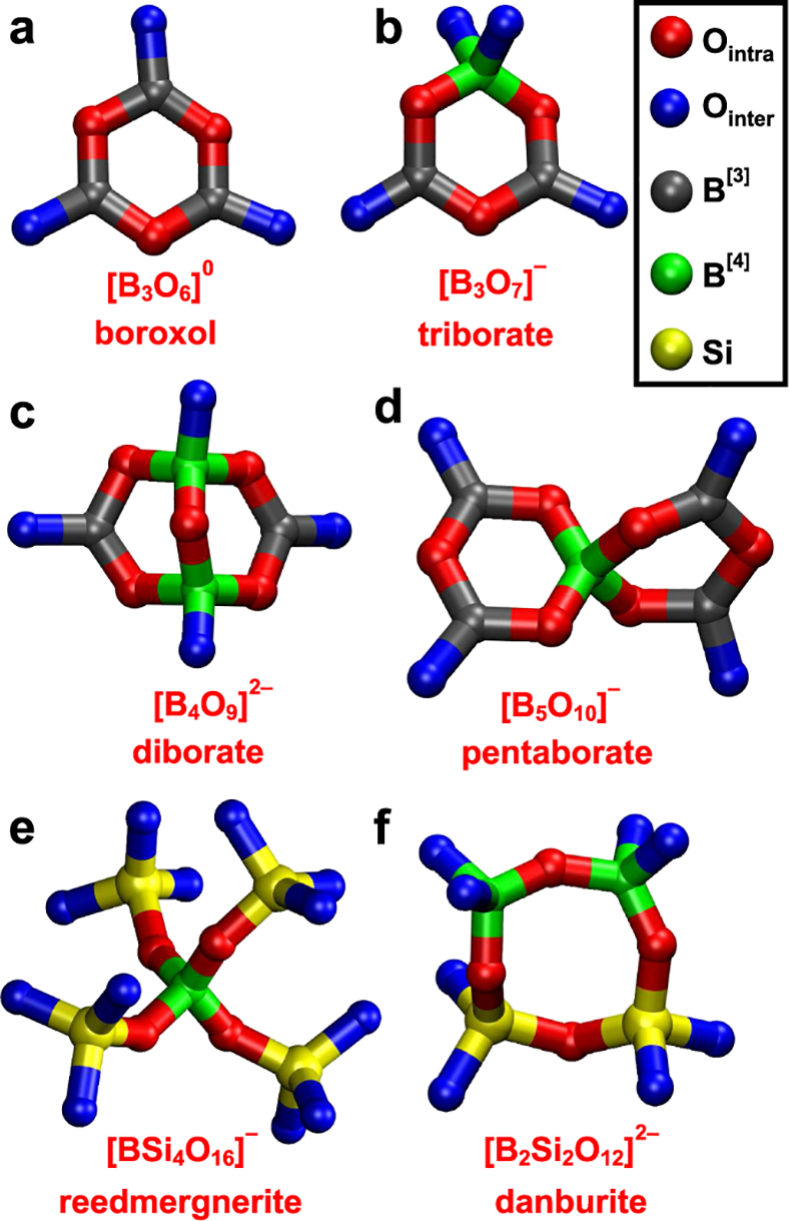
Depiction of (a–d) borate and (e, f) borosilicate superstructural units well-known to build the structures of crystalline borates and/or borosilicates, and proposed to also exist in the corresponding glassy structures.^1,20,44^ All O atoms are bridging (BO): O_intra_ refers to BO sites within each superstructural unit, whereas O_inter_ specifies atoms shared between adjacent units (not shown). Adapted from Edén^3^ with permission from Elsevier.

Vitreous B_2_O_3_ is well-known to comprise three BO_3_ groups in a boroxol ring, B_3_O_6_ (Figure 1a), along with a minor “non-ring” population of boroxol-ring-interlinking B^[3]^(NR) sites.^51−54^ For BS as well as NBO-bearing binary borate glasses, however, it remains unclear what precisely those “R” and “NR” structural motifs represent and, more importantly, whether *ad-hoc* assumed *two*-peak deconvolutions of the CT ^11^B^[3]^ NMR spectral region are adequate or too oversimplified. Most authors classified “ring” sites in borate/BS glasses strictly as boroxol moieties,^25−28,31,33,34,49,50,92^ while others attributed them to B^[3]^ sites present in *any* B^[3]^/B^[4]^ ring constellation,^29,30,32,93,94^ such as those depicted in Figure 1b–d. The B^[3]^(NR) environments in BS glass networks are typically identified by BO_3_ groups present in chain-like (i.e., non-ring) structural moieties featuring a significant interlinking with Si,^25−28,31,33,94^ but they have also been attributed to B^[3]^ sites in the BO_4_-bearing superstructural (ring) units shown in Figure 1b–d.^34^ Hence, analyses of the ^11^B^[3]^ NMR spectral region from BS glasses remain ambiguous. Our results do not offer any definite solution to this long-standing open question but will provide some qualitative constraints and guide future research directions that may possibly resolve the ambiguities.

The two ^11^B^[3]^(R) and ^11^B^[3]^(NR) environments of BS glasses manifest different isotropic chemical shift ranges of 16–18 ppm and 12–15 ppm, respectively (refs (25−28, 30−32, 34−36, 38, 49, 50, and 86)), which is observed in essentially any B-bearing phase, encompassing crystalline borates,^95^ vitreous B_2_O_3_,^51−54^ as well as binary *M*_2_O–B_2_O_3_^92,93^ and B_2_O_3_–SiO_2_^56,96^ glasses. For amorphous B_2_O_3_ and binary B_2_O_3_–SiO_2_ glasses, the deshielding has been traced to wider average interpolyhedral bond angles of θ̅(B^[3]^–O–B^[3]^) ≈ 135° ^52−54^ and θ̅(B^[3]^–O–B^[3]^/Si) ≈ 141°,^96^ respectively, around the B^[3]^(NR) sites relative to their B^[3]^(R) counterparts with average bond angles ∼ 120°. Hence, larger B^[3]^–O–Si bond angles may contribute to the progressively decreased ^11^B^[3]^ chemical shifts observed for increasing Si content in BS glasses and attributed to shielding effects from Si,^18,21,23,26,97^ as recently corroborated by density functional theory (DFT) calculations that verified a decrease in  for increasing number of ^11^**B**^[3]^–O–Si bonds.^89^ Moreover, in NBO-rich borate/BS glasses, ^11^B deshielding from NBO species at the BO_3_ groups may also account for the higher  values,^95,98,99^ regardless of the B^[3]^ participation in R or NR motifs (Section 5.3).

The hitherto perhaps most clear-cut assignments of the ^11^B^[3]^(R) and ^11^B^[3]^(NR) motifs—moreover deduced from complementary advanced MAS NMR experiments—may be those by Tricot^30^ for Pyrex glass (see Figure 8 of ref (30)). Both B^[3]^(R) and B^[3]^(NR) groups were concluded to interlink with “reedmergnerite” (Figure 1e) BS networks but differ in their contact modes: B^[3]^(NR) bonds directly via B^[3]^–O–Si linkages,^30^ whereas B^[3]^(R) participates in boroxol rings but also interlinks via B^[3]^–O–B^[4]^ bridges to B^[4]^(3Si) moieties, along previous inferences of Murakami et al.^29^ Howes et al.^34^ had earlier proposed a Pyrex-structure model related to that of Tricot,^30^ but involving both reedmergnerite^19,20^ and “danburite”^14,17,31^ (Figure 1f) motifs present in the B^[4]^/Si network, interleaved by boroxol rings (of B^[3]^(R) sites), via primarily triborate (Figure 1b) and pentaborate (Figure 1d) superstructural units.^1^ Hence, the “**B**^[3]^(NR)” sites resonating at ≈14 ppm also constitute “ring” sites in superstructural units comprising both B^[3]^/B^[4]^ coordinations.^34^

Although consistent with the experimental NMR results from the Si-rich and NBO-free Pyrex glass, however, it remains unclear how well the structural models of refs (29, 30, and 34) account for *B-richer* BS glasses. The 2Q–1Q ^11^B NMR spectrum from Pyrex revealed no B^[4]^–O–B^[4]^ linkages,^30^ whose nonetheless expected very low population would remain within the spectral noise in Figure 4 of ref (30), whereas B^[4]^–O–B^[4]^ bridges are readily identified from B-richer glasses.^41,49,50,67,68^ Notably, out of all superstructural units shown in Figure 1 and proposed by Howes et al.^34^ to build the Pyrex structure, only diborate groups involve a B^[4]^–O–B^[4]^ linkage (Figure 1c). Incidentally, eq 10 of ref (68) predicts a fraction of ≈3% B^[4]^–O–B^[4]^ linkages out of all B–O–B bridges in Pyrex glass, which agrees very well with the fraction of diborate groups deduced by Howes et al.^34^

## 4 Results

### 4.1 2Q–1Q and 2QF ^11^B NMR Results

**Figure 2:**
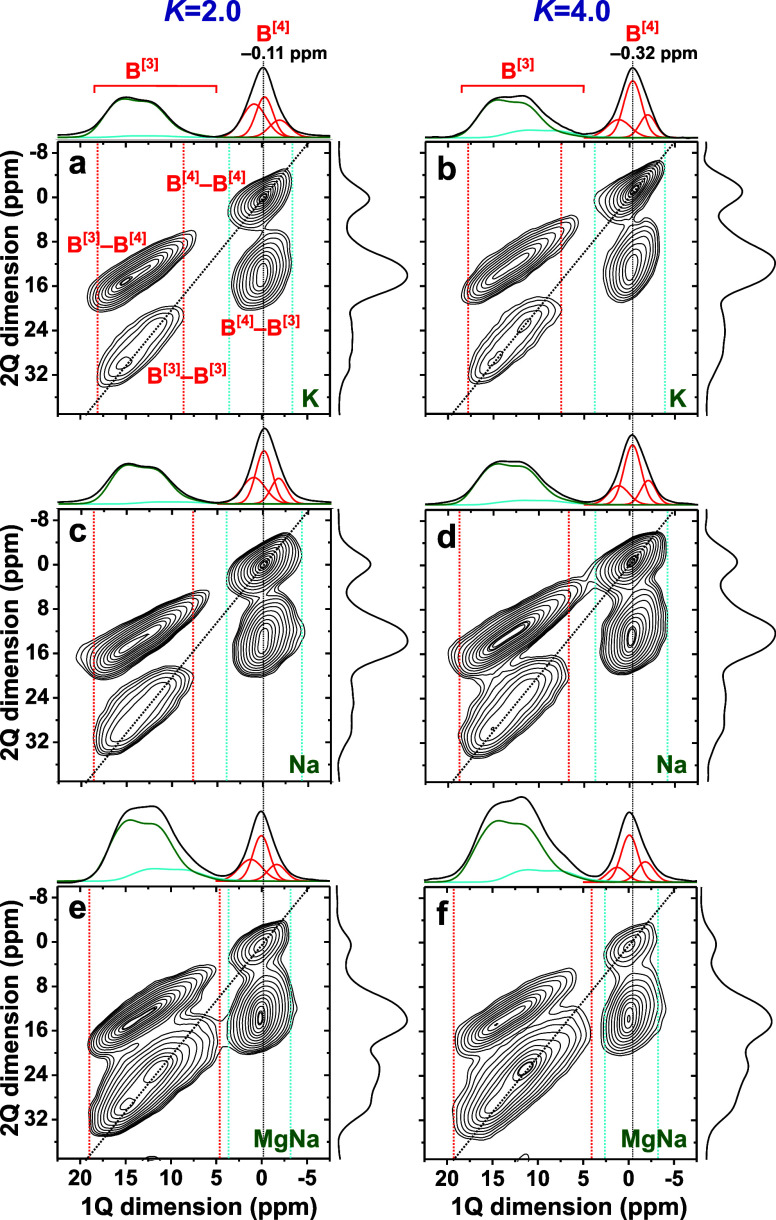
A selection of 2Q–1Q correlation ^11^B NMR spectra recorded at 14.1 T and 24.00 kHz MAS from BS glasses featuring *K* = 2.0 (left panel) and *K* = 4.0 (right panel) with (a, b) K^+^, (c, d) Na^+^, and (e, f) Mg^2+^/Na^+^ as glass-network modifiers. Projections along the 2Q and 1Q dimensions of the 2D NMR spectra are displayed at the right and at the top, respectively, where each 1Q projection is shown together with its B^[3]^(R) [green traces], B^[3]^(NR) [cyan traces], and B^[4]^(*m*Si) (red traces) peak components. The 2D NMR signals associated with each ^11^B^[3]^–O–^11^B^[3]^, ^11^B^[3]^–O–^11^B^[4]^, and ^11^B^[4]^–O–^11^B^[4]^ linkage are indicated in (a). The vertical red and cyan dotted lines mark the ^11^B shift extension of the ^11^B^[3]^–O–^11^B^[3]^ and ^11^B^[4]^–O–^11^B^[4]^ autocorrelation signals extending along the diagonal of slope 2 (dotted line). The spectra are presented with the lowest contour level of ≈5 % of the maximum 2D NMR peak amplitude.

Figure 2 shows a selection of 2Q–1Q correlation ^11^B NMR spectra acquired from the sets of *K* = {2.0, 4.0} BS glasses. As for a standard Bloch-decay ^11^B MAS NMR spectrum, the horizontal “1Q dimension” of the 2Q–1Q NMR counterpart comprises a broad high-shift () resonance from the lower-symmetry ^11^**B**O_3_ sites, along with a much narrower peak from the more symmetric ^11^**B**O_4_ tetrahedra, centered at  ppm. However, the 2D NMR spectrum provides direct insight into the various interlinked ^11^BO_*p*_–^11^BO_*q*_*pairs* present in the glass network, which are revealed along the vertical “2Q dimension” (Figure 2). Indeed, regardless of the precise network-modifier cation(s), all BS glasses comprise all three ^11^B^[3]^–O–^11^B^[3]^, ^11^B^[3]^–O–^11^B^[4]^, and ^11^B^[4]^–O–^11^B^[4]^ linkages, which resonate at the *sum* of 1Q shifts centered around , , and 2, respectively. Note that the two ^11^B^[3]^–^11^B^[3]^ and ^11^B^[3]^–^11^B^[4]^ correlations in Figure 2 appear as elongated “ridges” because the second-order quadrupolar broadening of the ^11^B^[3]^ signals extends along both spectral dimensions.^3,72^

We focus onward on the 1Q projection of each 2Q–1Q NMR spectrum, which is equivalent to a 2QF ^11^B NMR spectrum. A key feature of Figure 2 is that each ^11^B^[4]^ resonance detected in the 2QF NMR spectrum is a sum over both ^11^B^[4]^–^11^B^[4]^ and ^11^B^[4]^–^11^B^[3]^ 2D NMR peaks, while each 2QF ^11^B^[3]^ resonance is a superposition of ^11^B^[3]^–^11^B^[3]^ and ^11^B^[3]^–^11^B^[4]^. The pairs of red and cyan vertical guidelines in each 2Q–1Q NMR spectrum of Figure 2 mark the extent of the respective ^11^B^[3]^–^11^B^[3]^ and ^11^B^[4]^–^11^B^[4]^ “autocorrelation” ridge appearing along the spectral diagonal. The two narrow ^11^B^[4]^–^11^B^[3]^ and ^11^B^[4]^–^11^B^[4]^ correlation peaks overlap completely across the ±3 ppm range along the horizontal 1Q dimension. Likewise, the ^11^B^[3]^ spectral region with two broader ^11^B^[3]^–^11^B^[3]^ and ^11^B^[3]^–^11^B^[4]^ correlation ridges reveals that the latter spans a wider range of both higher and, in particular, lower, shifts than its ^11^B^[3]^–^11^B^[3]^ counterpart (Figure 2a–d). As may be verified from the more complete set of 2Q–1Q ^11^B NMR spectra presented in refs (67 and 68), those 2D spectral characteristics are general, except for BO_3_-dominated BS glasses, such as the Mg-bearing specimens (Figure 2e,f).

These trends offer the following important qualitative conclusions, which have direct implications for NMR peak assignments into {B^[4]^(*m*Si)} and {B^[3]^(R), B^[3]^(NR)} structural entities, as discussed further in Sections 4.2 and 4.3, respectively: *Both* B^[3]^(R) and B^[3]^(NR) structural motifs involve significant B^[3]^–O–B^[4]^ bonding, whereas *both* B^[3]^ and B^[4]^ species interlink with the B^[4]^(3Si) and B^[4]^(2Si) sites. Consequently, the results of Figure 2 supports previous proposals that even the highest-ppm ^11^B^[3]^ NMR spectral region attributed to “ring” sites *cannot solely* constitute B^[3]^–O–B^[3]^ motifs of boroxol rings but must also involve B^[3]^–O–B^[4]^ linkages.^29,30,32,93,94^

**Figure 3:**
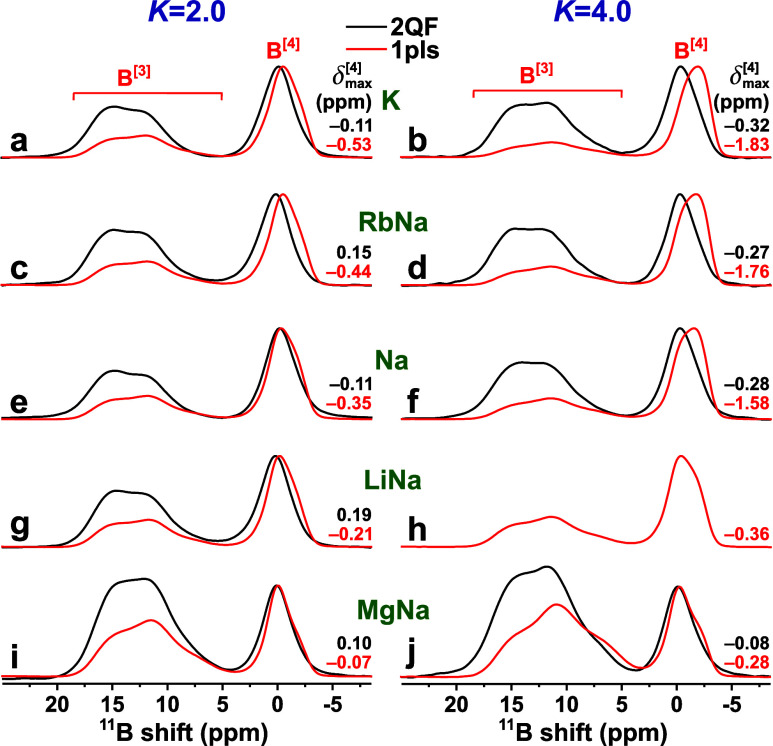
Projections of the 2Q–1Q ^11^B NMR spectra along the 1Q dimension (black traces; “2QF”) and ^11^B MAS NMR spectra obtained by single rf pulses (red traces; “1pls”) from the as-indicated *K* = 2.0 (left panel) and *K* = 4.0 (right panel) glass members. The black and red numbers at the rightmost spectral portions mark the shifts at the ^11^B^[4]^ NMR-peak maxima. No 2QF NMR result is available for the LiNa2.0 glass in (h).

Figure 3 contrasts each 2QF ^11^B MAS NMR spectrum with that of its directly excited Bloch-decay counterpart. Significant alterations are observed throughout the spectral region upon 2QF, manifested by a depletion of the low-ppm intensity of each ^11^B^[3]^ and ^11^B^[4]^ resonance region that consequently skews toward higher shifts. This feature stems from a reduced ^11^B^[*p*]^ shielding from Si atoms (Section 3), where we remind that the 2QF NMR spectrum (Figure 3) *only* comprises resonances from BO_*p*_ groups interlinked with *at least one* BO_*q*_ moiety. The emphasized high-ppm ^11^B^[3]^ resonance-intensities upon 2QF are more transparent in Figure S1, whereas Figure 3 conveys best the NMR peak-shape alterations observed for the ^11^B^[4]^ NMR spectral region, which reflects changes in the relative contributions from B^[4]^(*m*Si) structural motifs of each BS glass, whose ^11^B^[4]^(4Si) NMR-signal contributions are absent after 2QF application. The ^11^B NMR spectral deconvolutions discussed in sections 4.2 and 4.3 rationalize and quantify the following gross characteristics of Figures 3 and S1:

Regardless of the precise B and Si contents of the glass (i.e., its *K*-value) and which network-modifier species are present, the 2QF application emphasizes the ^11^B^[3]^ resonance intensity significantly at the expense of ^11^B^[4]^. Although partially reflecting a higher retention of the 2Q NMR signals involving ^11^B^[3]^ sites relative to those of ^11^B^[4]^ (Section 2.4), this trend suggests that the BO_3_ groups involve a comparatively larger fraction of bridges to other B^[*p*]^ sites than their BO_4_ counterparts that interlink with SiO_4_ groups to a higher extent. The most significant net ^11^B^[4]^ NMR peak-maximum () displacements upon 2QF result for the Si-richest *K* = 4.0 BS glasses (Figure 3), for which the fractional populations of the *lowest*-shift ^11^B^[4]^(4Si) environments are indeed expected to be largest, whereas much smaller  alterations are observed after 2QF for the B-richer *K* = 2.0 glasses. These trends apply regardless of the precise metal cation species in the BS structure, except for the Mg-bearing ones, which manifest markedly lower BO_4_ populations than any other glass. Moreover, the 2QF ^11^B^[4]^ NMR responses of the MgNa2.0 and, in particular, MgNa4.0 glasses remain much closer to their directly excited NMR counterparts (Figure 3i,j), which suggests comparatively fewer B^[4]^–O–Si linkages in the Mg-bearing glasses. The results of the MgNa*K* glasses are discussed separately in Section 4.4.

### 4.2 Double-Quantum-Assisted ^11^BO_4_ NMR-Peak Assignments

**Figure 4:**
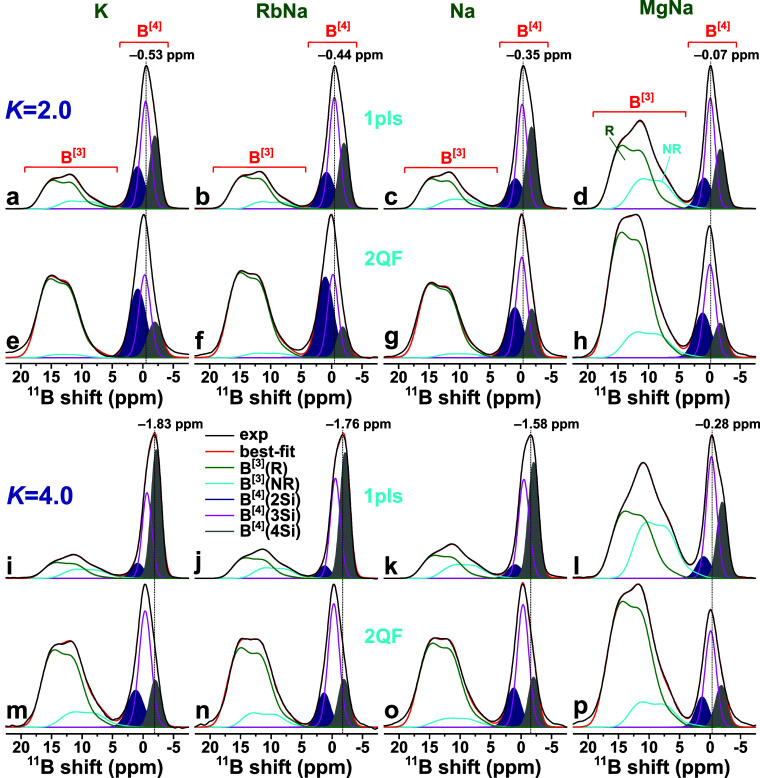
Experimental ^11^B NMR spectra (black traces) acquired at 14.1 T and 24.00 kHz MAS by either single pulses (“1pls”; a–d; i–l) or by 2QF (“2QF”; e–h; m–p) from the as-indicated BS glass series with *K* = {2.0, 4.0}. Red traces correspond to best-fit NMR spectra, whose peak components are identified by the legend in (j) and encompassing the following: “ring” (labeled “R”) and “non-ring” (“NR”) ^11^B^[3]^ NMR peaks, which are identified in (d), along with those from ^11^B(4Si), ^11^B(3Si), and^11^B(2Si) environments.

Figure 4 depicts the three-peak deconvolutions of the ^11^B^[4]^ resonance region in the absence and presence of 2QF, while Table 2 lists the corresponding sets of isotropic ^11^B^[4]^(*m*Si) chemical shifts, {δ_B_^[4]^(*m*Si)}, and fractional populations, {*x*_B_^[4]^(*m*Si)}. An inherent ambiguity of these spectral deconvolutions is to discriminate between the cases of (*A*) invariant δ_B_^[4]^(*m*Si) values but variable {*x*_B_^[4]^(*m*Si)} populations from that of (*B*) altered δ_B_^[4]^(*m*Si) shifts but (essentially) constant fractional populations, or (*C*) a combination of both. As discussed further in Section 5.2, however, significant population changes do occur among the coexisting B^[4]^(*m*Si) ensembles for increasing (average) CFS of the network modifiers.^27,38^

**Table 2:** Best-Fit ^11^B^[*p*]^ Chemical Shifts and Fractional Populations before and after 2QF^a^

		Fractional Population^b^					Isotropic Chemical Shift (ppm)				
	c	^11^B^[3]^		^11^B^[4]^(*m*Si)			^11^B^[3]^		^11^B^[4]^(*m*Si)		
Glass	1pls(2QF)	Ring	Non-ring	2Si	3Si	4Si	Ring	Non-ring	2Si	3Si	4Si
K2.0	0.660(0.463)	0.265(0.517)	0.075(0.020)	0.157(0.199)	0.299(0.175)	0.204(0.089)	17.95(18.54)	14.97(17.00)	1.00(0.97)	–0.37(−0.21)	–1.90(−1.90)
		0.779(0.963)	0.221(0.037)	0.237(0.430)	0.453(0.377)	0.309(0.192)					
RbNa2.0	0.634(0.435)	0.300(0.533)	0.066(0.031)	0.143(0.223)	0.315(0.154)	0.175(0.058)	17.82(18.26)	14.56(15.67)	1.00(1.20)	–0.32(−0.08)	–1.90(−1.70)
		0.820(0.944)	0.180(0.056)	0.226(0.513)	0.497(0.355)	0.277(0.132)					
Na2.0	0.635(0.472)	0.276(0.500)	0.089(0.028)	0.121(0.154)	0.284(0.217)	0.231(0.101)	17.88(17.90)	14.59(14.62)	1.00(1.10)	–0.12(−0.06)	–1.68(−1.70)
		0.757(0.947)	0.243(0.053)	0.190(0.327)	0.447(0.459)	0.364(0.214)					
LiNa2.0	0.597(0.435)	0.302(0.529)	0.101(0.036)	0.123(0.149)	0.291(0.222)	0.183(0.065)	17.82(18.10)	14.95(16.10)	1.05(1.50)	–0.04(0.02)	–1.63(−1.74)
		0.749(0.936)	0.251(0.064)	0.206(0.342)	0.488(0.510)	0.306(0.148)					
MgNa2.0	0.388(0.277)	0.409(0.592)	0.203(0.131)	0.077(0.089)	0.193(0.134)	0.118(0.053)	17.64(18.09)	14.44(15.54)	1.05(1.30)	0.03(0.10)	–1.62(−1.62)
		0.669(0.818)	0.331(0.182)	0.198(0.322)	0.499(0.485)	0.303(0.193)					
											
K4.0	0.713(0.423)	0.178(0.475)	0.109(0.102)	0.057(0.099)	0.254(0.239)	0.402(0.086)	17.50(17.90)	14.35(14.86)	1.00(1.30)	–0.58(−0.28)	–2.13(−1.96)
		0.621(0.823)	0.379(0.177)	0.079(0.233)	0.356(0.564)	0.565(0.203)					
RbNa4.0	0.686(0.402)	0.206(0.509)	0.108(0.089)	0.040(0.073)	0.289(0.240)	0.358(0.090)	17.41(18.44)	14.06(16.00)	1.30(1.40)	–0.48(−0.22)	–2.10(−1.90)
		0.657(0.852)	0.343(0.148)	0.058(0.181)	0.421(0.596)	0.522(0.222)					
Na4.0	0.650(0.414)	0.214(0.525)	0.135(0.061)	0.050(0.090)	0.283(0.237)	0.318(0.086)	17.46(18.15)	14.13(15.88)	1.07(1.30)	–0.40(−0.26)	–2.02(−2.02)
		0.613(0.895)	0.387(0.105)	0.076(0.217)	0.435(0.574)	0.489(0.208)					
MgNa4.0	0.338(0.247)	0.360(0.629)	0.303(0.124)	0.042(0.048)	0.182(0.138)	0.113(0.061)	17.52(18.30)	13.83(14.54)	1.13(1.37)	–0.17(−0.03)	–1.96(−1.80)
		0.543(0.835)	0.457(0.165)	0.125(0.196)	0.540(0.556)	0.334(0.248)					

a Fractional populations and isotropic ^11^B chemical shifts obtained by deconvoluting the ^11^B MAS NMR spectrum obtained directly by single-pulse excitation (“1pls”) and the projection along the 1Q dimension of the 2Q–1Q correlation 2D NMR spectrum (“2QF”; values within parentheses). Data uncertainties are listed in Table S2.

b Fractional populations normalized to a unity sum over all B^[3]^ and B^[4]^ species in the glass (top row for each glass). The row beneath lists the corresponding data normalized to a unity sum within each set of B^[3]^ and B^[4]^ populations:  and , where “R” and “NR” represent “ring” and “non-ring” sites, respectively.

c Net fractional population of the best-fit {*x*_B_^[4]^(*m*Si)} set: . The minor discrepancy to its  counterpart (“1pls”) listed in Table 1 stems partially from uncertainties in the spectral deconvolutions but primarily from the centerband ST ^11^B^[4]^ resonance intensity, which was not accounted for by the spectral deconvolution and leading to an overestimation of  by ≈ 0.03.

We first consider the deconvolutions of the Bloch-decay NMR spectra. The Si-rich *K* = 4.0 glass structures involve dominantly B^[4]^(4Si) and B^[4]^(3Si) environments that out of all tetrahedral sites account for 49–56% and 36–44%, respectively (Table 2), whereas the B-richer glasses manifest consistently higher B^[4]^(2Si) but lower B^[4]^(4Si) populations than their *K* = 4.0 counterparts, thereby rendering B^[4]^(3Si) motifs most abundant, and amounting to 45–50% of all B^[4]^(*m*Si) groups. In contrast to the previous spectral deconvolutions conducted for a larger set of *K* = 4.0 BS glasses reported by Lv et al.,^38^ we herein allowed the chemical shifts of the distinct-*m*^11^B^[4]^(*m*Si) moieties to vary slightly (within ±0.25 ppm). Within the caveats commented above, Table 2 reveals a weak chemical shift increase of 0.2–0.4 ppm for both δ_B_^[4]^(3Si) and δ_B_^[4]^(4Si) species for increasing CFS  of the glass-network modifier(s). The *K* = 2.0 glasses featured the most typical isotropic chemical shifts of {(2Si), (3Si), Si)} ≈ {1.0, −0.2, −1.7} (±0.2) ppm, whereas the respective δ_B_^[4]^(3Si) and δ_B_^[4]^(4Si) values of the *K* = 4.0 glasses are ≈0.20 ppm lower. Altogether, this amounted in chemical-shift separations of 1.6 ± 0.1 ppm between δ_B_^[4]^(3Si) and δ_B_^[4]^(4Si) for all Bloch-decay-derived NMR spectra, and somewhat more variable  differences of 1.0–1.5 ppm () and 1.3–1.8 ppm (*K* = 4.0).

Figure 4 evidences a consistent displacement of the net ^11^B^[4]^ resonance upon 2QF. Although that trend is indeed reflected in a minor deshielding by typically 0.1–0.3 ppm of each ^11^B^[4]^(*m*Si) site-ensemble, it derives primarily from a significant increase in the “apparent” best-fit  population while that of  is concurrently reduced. Naturally, the NMR-peak shape effects are most drastic for the Si-richer *K* = 4.0 BS glasses, for which B_B_^[4]^(4Si) environments are most abundant (Table 2). Likewise, the dominance of B_B_^[4]^(3Si) motifs in the *K* = 2.0 glasses with higher B contents readily account for the modest NMR peak maximum shift  alterations upon 2QF application in Figure 3. We underscore that the entire NMR peak intensity associated with the ^11^B^[4]^ resonance centered at the most negative shift (≈ –1.90 ± 0.2 ppm) would vanish upon 2QF application *if* it solely derived from ^11^B^[4]^(4Si) sites. The minor 5–10% of the integrated NMR intensities (out of all ^11^B^[*p*]^ sites) remaining after 2QF (Table 2) may partially stem from experimental artifacts and uncertainties in the deconvolutions but, most likely, also derive from an *incorrect* assumption about the structural origin of the NMR peak attributed solely to B^[4]^(4Si) sites. Hence, *some* of the NMR-signal intensity appearing  ppm may merely associate with ^11^B^[4]^(3Si) environments.

The detected ^11^B^[4]^ resonances of the horizontal projection of the 2Q–1Q NMR spectra constitute a sum from two correlation signals, ^11^B^[4]^–O–^11^B^[3]^ and ^11^B^[4]^–O–^11^B^[4]^, both of which overlap completely across the ^11^B^[4]^ resonance region (Figure 2), as is also evident from 2Q–1Q NMR results from other BS-based glasses.^41,49,50^ Hence, the overall narrow net resonance in MAS NMR spectra reflects shift perturbations of ^11^**B**^[4]^ from *all* of Si, B^[3]^, *and* B^[4]^ as neighbors, which is also corroborated by recent DFT-derived ^11^B chemical shifts.^89^ Although B^[4]^–O–^11^B^[3]^ linkages are strongly preferred^59,67,68,100^ and do dominate over B^[4]^–O–^11^B^[4]^ (Section 5.2), assumptions about Si and B^[3]^ species as sole bonding partners of B^[4]^ (refs (25−27, 30, 49, 50, and 86)) should be abandoned.

Yet, further guidance from advanced NMR experiments and computational modeling is required to enable more specific NMR-peak assignments of the various ^11^B^[4]^(*m*Si) environments and possibly disentangle the contributions from the variable numbers of B^[3]^/B^[4]^ neighbors of the **B**^[4]^(*m*Si) sites. Until then, we recommend to pragmatically pursue spectral deconvolutions with three {B^[4]^(*m*Si)} environments in general, where the 4 – *m* B^[4]^–O–B^[*p*]^ linkages (i.e., “B^[4]^–O–B”) are acknowledged to involve *either* B^[3]^*or* B^[4]^. At least from Si-rich glasses (nearly) devoid of NBO species, that strategy appears to offer reasonably accurate {*x*_B_^[4]^(*m*Si)} populations (Section 5.2), which likely reflects a partial error cancellation due to overlapping chemical shifts of the various ^11^**B**^[4]^–O–{B^[3]^, B^[4]^, Si} environments to justify the B^[3]^/B^[4]^ grouping of each fixed-*m*^11^B^[4]^(*m*Si) resonance.

### 4.3 Double-Quantum-Assisted ^11^BO_3_ NMR-Peak Assignments

We pursued the prevailing *ad hoc* two-component ^11^B^[3]^ NMR spectral-region deconvolution and contrast the results both with and without a 2QF stage (Figure 4), which shed further light onto the nature of the “ring” and “non-ring” ^11^B^[3]^ sites. We first consider the Bloch-decay-stemming NMR spectra and their deconvolutions, whose fractional populations quantitatively reflect the two trigonal ^11^B^[3]^(R) and ^11^B^[3]^(NR) types, within the caveats of the categorically simplified two-peak fitting. As expected,^25−28,31,33,94,96^ both ^11^B^[3]^(R) and ^11^B^[3]^(NR) environments manifest very similar quadrupolar products of 2.6–2.7 MHz throughout and mainly differ in their isotropic chemical shifts of 17.5–18 ppm and 14–15 ppm, respectively (Table 2). The most popular B^[3]^(NR) site-identification in the BS glass context involves BO_3_ groups in chain motifs with a significant degree of B^[3]^–O–Si bonding.^25−28,31,33,94^ The results herein support that notion, where the Si-richer *K* = 4.0 glasses reveal slightly lower δ_B_^[3]^(NR) shifts than their *K* = 2.0 counterparts, while exhibiting markedly higher NR:R ratios that roughly amount to 1:2, in contrast with those around 1:4 for the *K* = 2.0 glass members (Table 2).

The characteristics of a vast dominance of B^[3]^–O–B^[3]^/B^[4]^ bridges at the B^[3]^(R) sites are corroborated by the spectral alterations upon 2QF application (Figure 4): the high-ppm ^11^B^[3]^ resonance region stemming from B^[3]^(R) sites is significantly emphasized upon 2QF NMR application, but the isotropic chemical shift increases are more modest (typically within 0.6 ppm) than those of the Si-interlinked B^[3]^(NR) sites (1–2 ppm). We remind that the net ^11^B^[3]^ NMR peak observed in each 2QF MAS spectrum carries contributions from *both*^11^**B**^[3]^–O–B^[3]^*and*^11^**B**^[3]^–O–B^[4]^ linkages (Section 4.1), whose resonance spread across the entire ^11^B^[3]^ region. Hence, both B^[3]^(R) and B^[3]^(NR) ensembles feature linkages to *both* B^[3]^ and B^[4]^ sites, which yields two possibilities: Either B^[3]^(R) environments only participate in boroxol rings that are interlinked via “non-ring” BO_4_ tetrahedra. Given the comparable number of ^11^**B**^[3]^–O–B^[4]^ linkages relative to ^11^**B**^[3]^–O–B^[3]^ (as reflected in their respective 2D NMR signal intensities of Figure 2),^68^ however, it appears much more likely that most BO_3_ and BO_4_ groups share the same ring-structures, as in the superstructural units of Figure 1b–d. Hence, B^[3]^(R) is attributed to trigonal B sites mainly present in so-called “modified rings”.^29,30,32,93,94^

Because *only* resonances from ^11^**B**^[3]^(NR)–(OSi)_3_ motifs are removed identically by the 2QF process in these essentially NBO-free glasses (or ^11^**B**^[3]^(OSi)_*p*_(NBO)_3–*p*_ in NBO-bearing glasses), the ^11^B^[3]^(NR) signal fractions out of the entire 2QF ^11^B NMR intensity remain low but non-negligible, accounting for ≈5% and ≈15% for the *K* = 2.0 and *K* = 4.0 glasses, respectively (Table 2). Hence, the incomplete ^11^B^[3]^(NR) resonance-suppression implies that the “non-ring” BO_3_ groups *cannot solely* involve B^[3]^–O–Si linkages and that a significant fraction thereof must comprise at least one **B**^[3]^–O–B^[3]^/B^[4]^ linkage. We conclude that the B^[3]^(NR) sites in these BS glasses with  feature a *range* of linkages to *all* of the  network formers. Our qualitative findings from 2Q–1Q ^11^B NMR are aligned with earlier results from ^11^B/^29^Si double-resonance NMR by Wegner et al.,^33^ as well as by Du and Stebbins, who concluded from 3QMAS ^17^O NMR that ≈2/3 and ≲1/3 of all B^[3]^(NR) and B^[3]^(R) sites involve B^[3]^–O–Si linkages, respectively,^25,26^ along with a near-statistical B^[*p*]^/Si intermixing around the B^[3]^(NR) sites.^26^ Although there is a range of such B^[3]^(NR) sites with a variable number of B^[*p*]^/Si atoms in their second coordination spheres, the relative extents of B^[3]^–O–{B^[3]^, B^[4]^, Si} bonding cannot be inferred directly from our experimental data. Hence, spectral deconvolutions with additional ^11^B^[3]^ NMR peaks from further “non-ring” subsets are currently not warranted.

### 4.4 Mg-Bearing Glass Structures

As discussed further in Section 5.2, the strikingly different Bloch-decay and 2QF ^11^B NMR responses of the two Mg-bearing glasses shown in Figure 4 may be traced to their markedly higher B^[3]^ populations and accompanying larger number of B^[3]^–O–Si linkages at the expense of the typically prevailing B^[4]^–O–Si counterparts.^40^ However, if we disregard the reduction of all  values resulting from the lower net BO_4_ populations and merely focus on the populations normalized to a unity sum within each group of B^[3]^ and B^[4]^ coordinations, the B-richer MgNa2.0 glass does not reveal any striking differences to the other *K* = 2.0 glass members (Table 2). The Si-richer MgNa4.0 structure, on the other hand, manifests much fewer B^[4]^(4Si) moieties along with a significantly boosted B^[3]^(NR) abundance and *x*_B_^[3]^(NR):*x*_B_^[3]^(R) ratio. The 2Q–1Q ^11^B NMR spectra from both Mg-bearing glasses (Figure 2e,f) also manifest more intense ^11^B^[3]^–O–^11^B^[3]^ correlation ridges than all other glasses, suggesting that their networks may comprise a larger number of boroxol rings (Figure 1a). Altogether, the introduction of Mg in the Na4.0 glass amounts in a partial conversion of B^[3]^(R) sites with solely B^[3]^–O–{B^[3]^, B^[4]^} linkages into (*i*) B^[3]^(R) counterparts with emphasized B^[3]^–O–B^[3]^ interlinking at the expense of B^[3]^–O–B^[4]^ bridges, along with (*ii*) B^[3]^(NR) sites featuring B^[3]^–O–Si linkages. The latter observation agree with an increased (decreased) number of B^[3]^–O–Si (B^[4]^–O–Si) linkages inferred by a direct probing of the B^[*p*]^/Si intermixing via double-resonance ^11^B/^29^Si NMR experiments.^40^

## 5 Discussion

### 5.1 Role of Magnetic Field for Identifying ^11^B^[*p*]^ Environments

The precise external magnetic field utilized for ^11^B MAS NMR acquisitions sets some restrictions on how many, and which, ^11^B^[4]^(*m*Si) resonances may be detected and justified for subsequent spectral deconvolutions. It is well-known that ^11^B MAS NMR spectra obtained at *B*_0_ < 11.7 T reveal severely overlapping ^11^B^[3]^ and ^11^B^[4]^ signal regions; e.g., see refs (17, 28, 30, 42, and 101). While *B*_0_ = 11.7 T enables a near-complete ^11^B^[3]^/^11^B^[4]^ resonance separation, a significant overlap remains in the shift range of 1–4 ppm, as may be verified from the NMR spectra of refs (21, 28, 29, 31, 50, and 86). The typically minor ^11^B^[4]^(2Si) species resonate in that spectral range, which complicates the justification for their introduction into spectral deconvolutions. Although the resonance overlap is not fully suppressed at *B*_0_ = 14.1 T in all single-pulse NMR spectra of Figure 4, it appears to be the *minimum* field for sufficient NMR-signal separation to attempt deconvolutions into three {^11^B^[4]^(*m*Si)} signals, besides those from ^11^B^[3]^(R) and B^[3]^(NR) moieties.

Out of the plethora of existing reports invoking ^11^B MAS NMR spectral deconvolutions, encompassing those herein, only those from the very Si-rich and NBO-free Pyrex glass^28−30^ are truly unambiguous because high-field spectra (*B*_0_ ⩾ 18.8 T) proved that essentially all ^11^B^[4]^ spectral intensity is confined to two resonances, which thanks to the high *n*_Si_/*n*_B_ = 3.58 ratio of Pyrex may safely be attributed to B^[4]^(3Si) and B^[4]^(4Si) environments.^28−30^ Moreover, both Tricot^30^ and Prasad et al.^28^ deduced that  along with near-equal fractional populations of B^[4]^(4Si) and B^[4]^(3Si), which agreed excellently between the two studies once considering the data uncertainties. The ^11^B MAS NMR spectrum recorded at *B*_0_ = 21.8 T by Murakami et al.^29^ and decomposed into ^11^B^[4]^(3Si) and ^11^B^[4]^(4Si) NMR peaks also accords well with those of refs (28 and 30). Interestingly, a close inspection of Figure 1d of ref (29), however, reveals a very minor deviation between their experimental and best-fit spectra in the 1–2 ppm spectral region that likely reflects a minor unaccounted ^11^B^[4]^(2Si) resonance. The latter is even more evident from the ^11^B MAS NMR spectra shown in Figure 2 of Möncke et al.,^60^ and acquired from B-richer glasses at 18.8 T.

Although the ^11^B MAS NMR spectral resolution improves concurrently for increasing *B*_0_ across the narrow ^11^B^[4]^ resonance region, as well as enhancing the ^11^B^[3]^/^11^B^[4]^ frequency separation, the concomitant ^11^B^[3]^ resonance-narrowing may *compromise* the discrimination among B^[3]^(R) and B^[3]^(NR) environments, *unless* sufficiently high fields (>20 T) are employed to significantly suppress the second-order quadrupolar broadening.^29^ Hence, if two distinct magnetic fields are available, numerical deconvolutions of NMR spectra recorded at both fields are recommended.^28−32^

### 5.2 Glass Composition Constraints on the {B^[4]^(*m*Si)} Set

Here, we discuss the strong bearings of the B^[4]^(*m*Si) populations of the BS glass network on its (*I*) *n*_Si_/*n*_B_ molar ratio (i.e., on the *K*-value), (*II*) {, } speciation, and (*III*) NBO content. The number of coexisting B^[4]^(*m*Si) groups, however, is *a priori* unknown because the underlying preferences of the B^[4]^ sites to interlink with Si, B^[3]^, and B^[4]^ are not known quantitatively by experiments, which underlies why the current choices of either two- or three-peak spectral deconvolutions are rarely justified. Here we make an attempt with guidance from MD simulations.

Table 3 compares each NMR-derived {*x*_B_^[4]^(*m*Si)} set of the Na2.0 and Na4.0 glasses and their Mg-bearing counterparts with those obtained from atomistic MD simulations available from the glass models presented by Lv et al.^40^ Each set of experimental and modeled populations is normalized to a unity sum and is contrasted with those predicted for an unconstrained statistical B^[4]^–O–{Si, B^[3]^, B^[4]^} linkage formation, calculated from the {*x*_*F*_} fractions of Table 3 along with equal bonding preferences, and assuming the absence of NBO. That assumption is well justified by the very close statistical populations deduced from the MD-generated glass models when rigorously accounting for their actual average number of BO atoms per *F* atom (Table 3). For a statistical B^[4]^–O–*F* linkage formation, all *K* = {2.0, 4.0} glass structures would comprise four distinct-*m* B^[4]^(*m*Si) moieties with non-negligible populations [i.e., all but B^[4]^(0Si)], revealing average numbers *m̅* ≈ 2.1 and *m̅* ≈ 2.8 for the *K* = 2.0 and *K* = 4.0 glasses, respectively. The three-peak deconvolutions of the NMR spectra, however, yielded significantly higher values of *m̅* = 3.2 and *m̅* = 3.4 for the Na2.0 and Na4.0 glasses, respectively, whereas the modeled *m̅* values are intermediate of the experimental and statistical counterparts. A more disordered modeled *m*-distribution than that inferred by NMR is reminiscent of the BO/NBO partitioning among the SiO_4_ groups in silicate glasses.^102−104^

**Table 3:** NMR- and MD-Derived Fractional Populations of B^[4]^(*m*Si) Groups^a^

		Molar Fractions				B^[4]^(*m*Si) Fractions				
Glass		*x*_Si_			*m̅*^b^	0	1	2	3	4
Na2.0	NMR	0.500	0.182	0.318	3.18(2.10)	0.000(0.051)	0.000(0.226)	0.190(0.374)	0.446(0.274)	0.364(0.075)
	MD	0.500	0.227	0.273	2.50(2.12)	0.019(0.049)	0.116(0.220)	0.358(0.372)	0.365(0.280)	0.142(0.079)
					c(2.14)	c(0.047)	c(0.215)	c(0.371)	c(0.285)	c(0.082)
										
MgNa2.0	NMR	0.500	0.306	0.194	3.11(2.17)	0.000(0.044)	0.000(0.209)	0.198(0.370)	0.499(0.291)	0.303(0.086)
	MD	0.500	0.323	0.177	2.36(2.18)	0.026(0.043)	0.156(0.206)	0.354(0.369)	0.358(0.294)	0.106(0.088)
					c(2.23)	c(0.038)	c(0.192)	c(0.365)	c(0.308)	c(0.097)
										
Na4.0	NMR	0.667	0.117	0.216	3.41(2.75)	0.000(0.010)	0.000(0.084)	0.076(0.278)	0.435(0.406)	0.489(0.222)
	MD	0.667	0.157	0.176	3.06(2.78)	0.002(0.009)	0.040(0.080)	0.176(0.270)	0.458(0.409)	0.324(0.232)
					c(2.79)	c(0.008)	c(0.078)	c(0.268)	c(0.410)	c(0.236)
										
MgNa4.0	NMR	0.667	0.220	0.113	3.21(2.82)	0.000(0.008)	0.000(0.072)	0.125(0.259)	0.540(0.414)	0.335(0.248)
	MD	0.667	0.233	0.100	2.89(2.83)	0.005(0.007)	0.054(0.071)	0.242(0.257)	0.448(0.413)	0.251(0.251)
					c(2.87)	c(0.006)	c(0.064)	c(0.246)	c(0.418)	c(0.266)

a Fractional populations of B^[4]^(*m*Si) groups, (*m*Si), obtained either by fitting the Bloch-decay ^11^B MAS NMR spectra (“NMR”) or calculated by MD simulations (“MD”).^40^ The values in parentheses represent populations predicted from an unconstrained statistical distribution of B^[4]^–O–Si/B^[*p*]^ linkages, calculated from the binomial expression , with 0 ⩽ *m* ⩽ 4 and . The {, } data of the glass was employed, with borate speciations extracted either from the glass models or the [fit] data listed in Table 2. The minor degree of *F*–NBO bonding was ignored.

b Average number of B^[4]^–O–Si bonds calculated from (*m*Si).

c Statistical {(*m*Si)} results calculated from a binomial distribution with , where {, , } is the set of MD-derived average number of *F*–BO bonds at the respective {Si, B^[3]^, B^[4]^} ensembles in the glass model.^68^ The very good agreement with the statistical populations obtained when ignoring the small number of *F*–NBO bonds (data on line above) justifies their use for both the experimental and modeled data.

Both modeled and experimental {} data sets of Table 3 suggest significant {B^[4]^(2Si), B^[4]^(3Si), B^[4]^(4Si)} populations throughout all glass structures. The previously^25−27^ and herein observed markedly stronger B^[4]^–O–Si interlinking than that of a statistical B^[4]^/*F* intermixing, may be rationalized from the widely differing {*x*_*F*_} abundances and propensities of Si, B^[3]^, and B^[4]^ to interlink with BO_4_ groups^40,68^ (Table S3). Although B^[4]^–O–B^[3]^ linkages are preferred over B^[4]^–O–Si, *P*(B^[4]^–O–B^[3]^) > *P*(B^[4]^–O–Si), the markedly higher Si abundance coupled with its 4/3 larger number of available BO sites, promotes B^[4]^–O–Si bridges over B^[4]^–O–B^[3]^, and notably so B^[4]^–O–B^[4]^, which are preferentially avoided due to *P*(B^[4]^–O–B^[4]^) ≈ 0.5.^40,68^ The deviation from unity of the preference factor *P*(B^[4]^–O–*F*) marks the degree of preference [*P*(B^[4]^–O–*F*) > 1] or reluctance [*P*(B^[4]^–O–*F*) > 1] for creating B^[4]^–O–*F* linkages. The {*x*_*F*_} and {*P*(B^[4]^–O–*F*)} entities together rationalize the dominance of ≈2.5 and ≈3 Si atoms in the second coordination sphere of the B^[4]^ sites in the respective *K* = 2.0 and *K* = 4.0 MD-derived glass models (Table 3).

**Figure 5:**
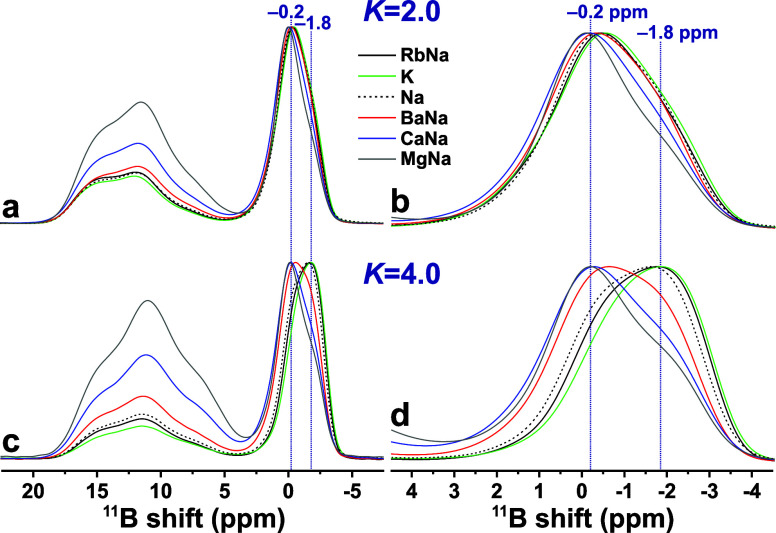
Bloch-decay ^11^B NMR spectra recorded at 14.1 T and 24.00 kHz MAS from glasses with network modifiers specified in the legend and featuring (a, b) *K* = 2.0 and (c, d) *K* = 4.0, where (a, c) display the entire CT NMR spectral region and (b, d) are zooms over the ^11^B^[4]^ resonances.

For decreasing (average) CFS of the network modifier(s) across the *K* = 4.0 glass branch, both the net BO_4_ population and the B^[4]^/Si intermixing *increase* concurrently, where Lv et al.^40^ demonstrated a linear correlation between the decreasing fraction of B^[4]^–O–Si linkages for increasing *M*^*z*+^ CFS. The strong interplay between the three {*P*(B^[4]^–O–*F*)} preferences, the *n*_Si_/*n*_B_ ratio of the BS glass, and its {B^[3]^, B^[4]^} speciation is reflected in the ^11^B MAS NMR spectra shown in Figure 5 for a larger number of ternary *M*_2_O–B_2_O_3_–SiO_2_ and quaternary *M*_(2)_O–Na_2_O–B_2_O_3_–SiO_2_ glasses with different alkali and alkaline-earth metal cations.^10^ For increasing (average) CFS of the *M*^+^/*M*^2+^ cation(s), the fractional BO_3_ populations are increased at the expense of BO_4_, as is most evident from the full ^11^B CT MAS NMR spectral regions displayed in Figure 5a,c.

We onward focus on the ^11^B^[4]^ NMR peak shapes shown in Figure 5b,d, where strikingly different responses are observed between the two glass branches: the Si-richer glasses manifest a conversion from a dominance of ^11^B^[4]^(4Si) NMR-signal intensity observed from the low-CFS alkali-based glasses to progressively more intense ^11^B^[4]^(3Si) resonances for increasing *M*^2+^ CFS. Indeed, the NMR spectra of the MgNa4.0 and CaNa4.0 specimens are closer to those of the B-richer glasses shown in Figure 5b. Their common spectral signature, peaking around the typical shift of ^11^B^[4]^(3Si) groups, is attributed to higher BO_3_ populations (Table 3) that boost the number of B^[4]^–O–B^[3]^ bridges. Likewise, the much larger B^[4]^(4Si) populations of the *M*Na4.0 glasses with low-CFS *M*^+^ cations stem from their comparably low B^[3]^ abundances, which restrict formation of the most preferred B^[4]^–O–B^[3]^ linkages,^59,68,100^ while there is a *reluctance* for forming the otherwise statistically favored B^[4]^–O–B^[4]^ bridges. Hence, B^[4]^–O–Si linkages become boosted due to their stronger formation preference than B^[4]^–O–B^[4]^ along with the high Si content of the glass (Table S3). Note that whenever , B^[4]^–O–B^[4]^ bridges *must* exist in any NBO-free *M*_(2)_O–B_2_O_3_ glass, while B^[4]^–O–Si linkages are effectively promoted in the BS glass analog.

The results of Table 3 and Figure 5 underscore the strong bearings from both the  ratio of the glass and its BO_3_ population on the precise {B^[4]^(*m*Si)} speciation. Notably, the B^[3]^ reservoir of the MgNa4.0 glass () is close to that of its B-richer Na2.0 counterpart (), as is mirrored in similar average numbers *m̅* ≈ 3.2 of B^[4]^–O–Si linkages observed by NMR (Table 3). Owing to a previously discussed MD-derived increasing preference for *F*–NBO bonding according to B^[4]^ ≪ Si < B^[3]^,^40,41,68,100^ the number of B^[4]^–O–Si linkages grows for increasing NBO content of the BS glass, which is verified by the {} sets obtained from Na^+^ and Na^+^/Ca^2+^ glass models (data not shown) but remains to be shown experimentally.

We conclude by noting that although the ^11^B^[4]^(2Si) resonance remains swamped by the dominant ^11^B^[4]^(3Si) NMR peak in Figures 3 and 4, three ^11^B^[4]^(4Si), ^11^B^[4]^(3Si), and ^11^B^[4]^(2Si) signal components were required in our deconvolutions. Besides the 3QMAS ^11^B NMR results of Du and Stebbins,^26^ minor ^11^B^[4]^(2Si) NMR intensities are hinted (but not commented) in other ^11^B NMR spectra in the literature.^29,60^ Non-negligible B^[4]^(1Si) populations are moreover anticipated for BS glasses with *K* < 2.0, notably for those featuring high BO_3_ populations (*vide supra*). Attempting such three-/four-peak deconvolutions from the set {^11^B^[4]^(3Si), ^11^B^[4]^(2Si), ^11^B^[4]^(1Si), ^11^B^[4]^(0Si)}, however, requires further knowledge of the chemical-shift dependence on *m*. That demands additional work on δ_B_^[4]^(*m*Si) predictions by DFT calculations,^43,89^ along with ^11^B MAS NMR experiments on BS glasses with variable *n*_Si_/*n*_B_ ratios at very high field (*B*_0_ > 20 T), which is likely also needed for enabling sufficient discrimination among the various coexisting ^11^B^[3]^, ^11^B^[4]^(0Si), and ^11^B^[4]^(1Si) resonances, as well as shedding light on the current enigma concerning the unknown (in)variance of the  shifts for glasses incorporating different glass-network modifiers (Section 4.2).

### 5.3 Remaining Challenges: NBO-Rich Glasses

With a few recent exceptions,^49,50,84^ previous ^11^B NMR spectral deconvolutions predominantly concerned BS-based glasses with low NBO contents. Analyses of glasses with non-negligible NBO populations must account for the bearings from B^[3]^–NBO bonds and possibly also B^[4]^–NBO. Here, we review the current state of affairs and identify future research directions required for *potentially* reaching more realistic ^11^B NMR spectral deconvolutions of NBO-rich BS-based glasses. We note that although ^17^O NMR may distinguish NBO bonding to Si and B,^24,64^ it cannot discriminate between the two B^[3]^–NBO and B^[4]^–NBO scenarios (Section 1). ^11^B/^17^O double-resonance NMR is the most direct experimental approach to probe both B^[3]^–NBO (Section 5.3.1) and B^[4]^–NBO bonding, which is currently being explored. Below we focus on the effects of ^11^B^[*p*]^ coordination upon NBO coordination.

#### 5.3.1 B^[3]^–NBO Bonding

NBO-for-BO substitutions in the first coordination sphere of a network-forming *F* site are well-known to increase its chemical shift, as is long known to hold for the ^29^Si and ^31^P nuclides.^2−4^ increases concurrently by a few ppm for each B^[3]^–BO → B^[3]^–NBO bond replacement.^98,99,105^ For NBO-bearing binary borate glasses, the ^11^B^[3]^ NMR spectral regions are traditionally analyzed solely in terms of distinct B^[3]^(*q*NBO) environments (featuring *q* B^[3]^–NBO bonds),^95,98,99,105,106^ rather than the B^[3]^(R) and B^[3]^(NR) counterparts. The discrimination among distinct-*q*^11^B^[3]^(*q*NBO) resonances is not primarily based on their isotropic chemical shifts but rather on the distinct ranges of the quadrupolar asymmetry parameters. Here, the formally axially symmetric efg tensor () associated with ^11^B^[3]^(0NBO) sites typically reveals , whereas those with 1–2 B^[3]^–NBO bonds manifest larger asymmetry parameters .^95,98,99^ Early wide-line ^11^B NMR on static BS glass powders enabled estimations of the ^11^B^[3]^(0NBO) population relative to its NBO-bearing (*q* ⩾ 1) counterparts,^19,20^ but convincing MAS NMR spectral deconvolution analyses are still lacking.

Saini et al.^84^ analyzed the ^11^B^[3]^ NMR signal regions from a large series of BS glasses with variable B and NBO contents by employing the prevailing two-peak deconvolution into B^[3]^(R) and B^[3]^(NR) contributions, where the latter was assigned to non-ring B^[3]^ sites interlinking with Si. For the NBO-bearing glasses, however, the “B^[3]^(NR)” sites were on the basis on their high best-fit  values of 0.6–0.7 attributed to B^[3]^(1NBO) environments,^84^ implying that all B^[3]^–NBO bonds occur at the non-ring/Si-interlinked BO_3_ moieties, which would then coexist with B^[3]^(R) sites without bonds to NBO anions. It is difficult to estimate both  values of the B^[3]^(R) and B^[3]^(NR) species accurately, which already represent simplified structural entities across a potentially much more complex BO_3_ ensemble with variable numbers of both BO/NBO bonds and {Si, B^[3]^, B^[4]^} neighbors, and thereby also  values. It therefore remains unclear how to merge the two hitherto proposed but distinctly different B^[3]^ classifications of B^[3]^(*q*NBO) motifs devoid of B^[3]^–O–Si bonds with the prevailing B^[3]^(R)/B^[3]^(NR) moieties introduced for NBO-free borate/BS glasses.

#### 5.3.2 B^[4]^–NBO Bonding

**Figure 6:**
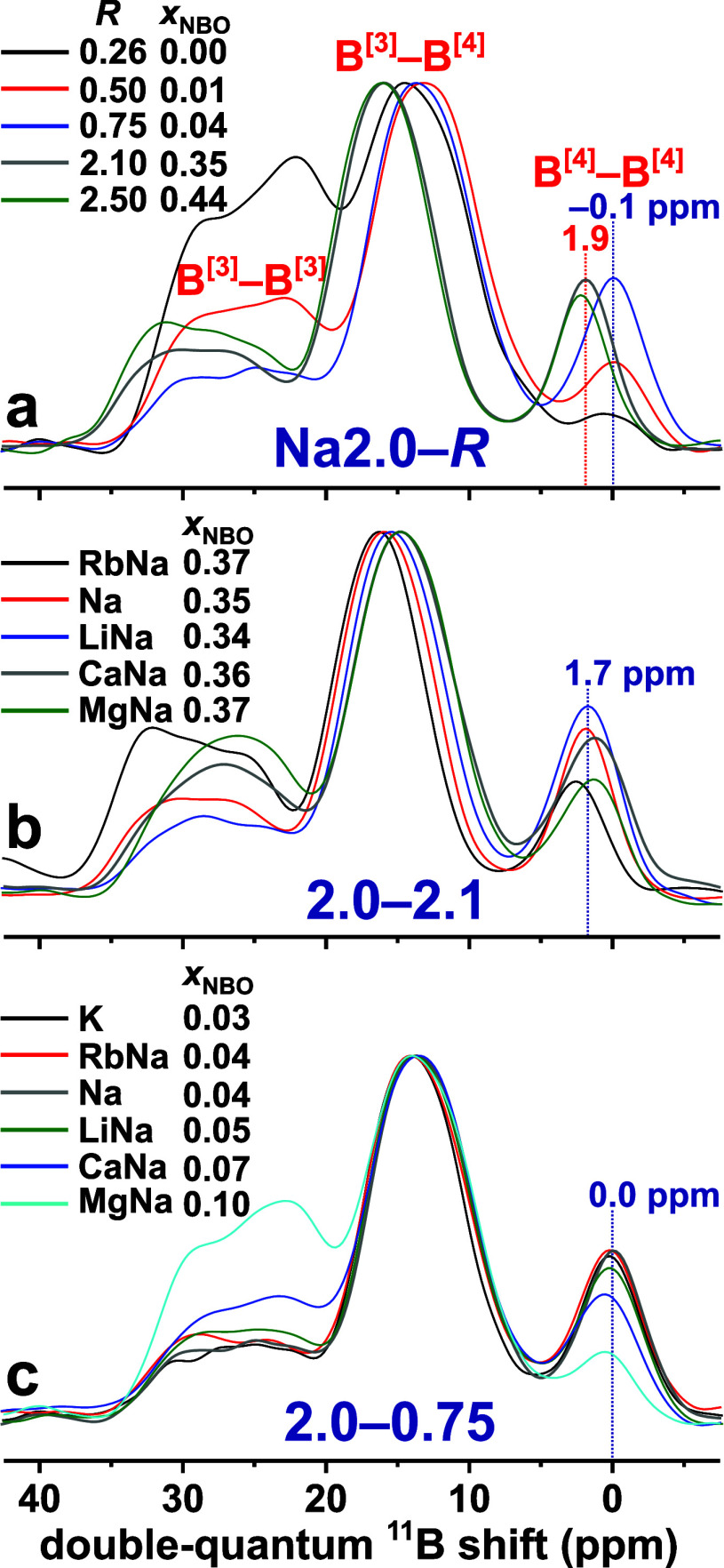
Projections along the 2Q dimension of 2Q–1Q ^11^B NMR spectra from *K*–*R* BS glasses, with *R* given by Equation 3: (a) Na2.0–*R* with *R* increasing (see legend), (b) 2.0–2.1, and (c) 2.0–0.75. Each legend specifies the NBO content (*x*_NBO_) of the glass, along with its network modifier(s) in (b, c). As indicated in (a), each 2Q spectrum comprises three groups of 2Q signals from ^11^B^[3]^–O–^11^B^[3]^ (left), ^11^B^[3]^–O–^11^B^[4]^ (middle), and ^11^B^[4]^–O–^11^B^[4]^ (right) linkages.

As for the presence of B^[4]^–O–B^[4]^ linkages of BS-based glasses (Section 4.2), potential chemical-shift effects from ^11^**B**^[4]^–NBO bonds also remain ignored in the literature. Notwithstanding their expected absence in all but (very) NBO-rich glasses,^39,41,67,68,88,89^ their potential presence would severely complicate ^11^B MAS NMR spectral deconvolutions from NBO-rich BS glasses, where the NMR parameters of the distinct-*m*^11^B^[4]^(*m*Si) moieties are modified depending on the presence/absence of NBO anions at the tetrahedron. While such ambiguities of the ^11^B^[4]^(*m*Si) chemical shifts from B^[4]^–NBO bonding are irrelevant for the present analysis of low-NBO glasses, the following observations are noteworthy:

(*i*) DFT calculations predict increased ^11^B^[4]^ chemical shifts upon NBO coordination.^89^ (*ii*) Borophosphosilicate glasses with high Si and NBO contents reveal consistently slightly higher  values (by ≈0.5 ppm) than their NBO-poor counterparts,^39,43^ which might stem from a minor degree of B^[4]^–NBO bonding. (*iii*) 2Q–1Q correlation ^11^B NMR experiments of NBO-rich BS glasses *also* suggest slightly higher ^11^B^[4]^ shifts relative to their NBO-poor counterparts. That feature is most evident from the 2Q projections of 2Q–1Q ^11^B NMR spectra shown in Figure 6a, which were recorded from a series of *R*Na_2_O–B_2_O_3_–2.0SiO_2_ glasses with increasing Na_2_O content *R*, where we employ the more general *MK*–*R* or *M*Na*K*–*R* glass notation of refs (40 and 68). The NBO contents are negligible in all *R* ⩽ 0.75 glasses but significant for the *R* = {2.1, 2.5} counterparts (the 2D NMR spectra are displayed in Figure 2 of Lv et al.^68^). Notably, the NBO-free Na BS glasses reveal near-constant peak-maxima shifts of ≈0.0 ppm for the ^11^B^[4]^–O–^11^B^[4]^ correlation peak, whereas those of the two Na2.0–2.1 and Na2.0–2.5 glasses are markedly higher by ≈2 ppm. Likewise, the 2Q peak maxima observed from the 2.0–2.1 glass series with *x*_NBO_ ≈ 0.36 shown in Figure 6b are consistently ≈1.7 ppm higher than their NBO-free 2.0–0.75 counterparts displayed in Figure 6c.

Although both trends (*ii*) and (*iii*) evidence a minor but consistent ^11^B^[4]^ deshielding for elevating NBO contents, further studies are needed for confirming that they indeed stem from B^[4]^–NBO bonds and not from other structural changes, such as ^11^B deshielding from altered bond-angle distributions around the tetrahedral sites, where decreased B^[4]^–O–*F* bond angles are also expected to increase .^95^

## 6 Conclusions

The “spectral editing” features of double-quantum filtration as probed via the horizontal projection of a 2Q–1Q ^11^B 2D NMR spectrum offered qualitative constraints on the ^11^B^[3]^ and ^11^B^[4]^ resonance assignments concerning their degrees of intermixing with the SiO_4_, BO_3_, and BO_4_ network groups in two BS glass branches with variable network modifiers but (very) low NBO contents and fixed molar ratios *n*_Si_/*n*_B_ of 1.0 (*K* = 2.0) and 2.0 (*K* = 4.0). 2QF suppresses ^11^B^[*p*]^ resonances from sites without any direct O linkage to another B neighbor, whereas those from ^11^B^[*p*]^ sites with several B neighbors become emphasized.

The 2Q–1Q correlation NMR results show conclusively that the BO_4_ groups may interlink with *any* {Si, B^[3]^, B^[4]^} species but the correspondingly decreasing preference factors *P*(B^[4]^–O–B^[3]^) > *P*(B^[4]^–O–Si) ≫ P(B^[4]^–O–B^[4]^),^40,67,68^ along with the *n*_Si_/*n*_B_ molar ratio and {, } speciation, strongly affect the B^[4]^–O–{Si, B^[3]^, B^[4]^} intermixing. Hence, for Si-rich glasses with dominantly BO_4_ groups—such as all *K* = 4.0 glasses herein with low-CFS alkali-metal cations—limits the formation of the *most preferred* B^[4]^–O–B^[3]^ linkages and effectively boosts the number of B^[4]^–O–Si bridges, which are strongly preferred over B^[4]^–O–B^[4]^. Hence, as observed previously for similar glass compositions,^25−27^ our ^11^B MAS NMR spectral deconvolutions revealed that B^[4]^–O–Si linkages dominate and implying on average ≈3.1 and ≈3.4 Si atoms around the B^[4]^ sites in the *K* = 2.0 and *K* = 4.0 glasses, respectively. However, because the borate speciation depends strongly on the field strength of the network modifiers and the BO_3_ abundance increases concurrently with the *M*^*z*+^ CFS,^10,38^ both the ^11^B^[4]^ MAS NMR spectrum and the average number of B^[4]^–O–Si linkages of the MgNa4.0 glass are much closer to those of the B-richer *M*Na2.0 glasses than its Si-rich sister glasses.

For the hitherto most common range of BS glasses with comparable Si and B contents and *low* NBO contents, such as those with molar fractions 1 ⩽ *n*_Si_/*n*_B_ ⩽ 2 considered herein, we recommend deconvoluting the ^11^B^[4]^ resonance region with three peak components, i.e., {B^[4]^(2Si), B^[4]^(3Si), B^[4]^(4Si)}, where B^[4]^(*m*Si) implies 4 – *m* bonds to “B”, which may constitute either of B^[3]^ and B^[4]^ but with the former dominating. The hitherto most popular two-peak deconvolution that only accounts for B^[4]^(3Si) and B^[4]^(4Si) sites, however, will significantly overestimate the degree of B^[4]^–O–Si linkages at the expense of B^[4]^–O–B^[*p*]^ for comparatively B-rich BS-based glasses (*n*_Si_/*n*_B_ < 2). It is therefore only justified for NBO-free but *very* Si-rich glasses, such as Pyrex.^28−30^

Concerning the ^11^B^[3]^ NMR spectral region, we employed the prevailing but rather arbitrary two-component deconvolution into resonances from B^[3]^(R) (“ring”) and B^[3]^(NR) (“non-ring”) sites. 2QF application strongly emphasizes the NMR signal intensities from the B^[3]^(R) sites, implying that they involve very few (if any) bonds to Si but comparable amounts of B^[3]^–O–B^[3]^ and B^[3]^–O–B^[4]^ linkages, consistent with BO_3_ and BO_4_ groups that coexist in superstructural “ring” units. Yet a non-negligible fraction of the B^[3]^ sites may form boroxol groups, notably so in glasses with dominant BO_3_ populations, such as in Mg-bearing glasses (Figure 2). The B^[3]^(NR) sites, on the other hand, involve a significant B^[3]^–O–Si bonding, in particular in the Si-rich MgNa4.0 glass, as verified from their markedly lower NMR-signal intensities resulting after 2QF. Combining the NMR results herein and in ref (40) conclusively shows that the B^[3]^(NR) sites feature linkages to all of B^[3]^, B^[4]^, and Si, where the latter are likely dominating, along previous findings.^25,26,33^

Although these two- and three-NMR-peak representations of the respective B^[3]^ and B^[4]^ environments in BS glasses are obviously oversimplified, they currently appear to be the only option for even attempting to characterize the *F* = {Si, B^[3]^, B^[4]^} intermixing around the tri- and tetragonal B sites in BS-based glasses via routine ^11^B MAS NMR experiments. In general, however, such multiparameter spectral-deconvolution analyses may *at best* provide semiquantitative site populations. More reliable and accurate data are only expected in limited cases, such as for ^11^B MAS NMR spectra recorded at high magnetic fields (⩾18.8 T) from very Si-rich glasses.^28−30^ We also underscore that these caveats concern essentially NBO-free BS glasses, while we discourage even attempting spectral deconvolutions from glasses with significant NBO contents. Circumstantial evidence does suggest that ^11^B^[4]^ chemical shifts are consistently higher in NBO-rich glasses. Yet additional insight from complementary spectroscopic techniques and advanced ^11^B/^17^O MAS NMR experimentation, along with structure/chemical-shift investigations by DFT calculations, is required to gain further insight. This is currently underway in our laboratory.

## Supporting Information Available

Tables with additional data of CFS values, best-fit parameters and data uncertainties of the spectral deconvolutions, and MD-derived preference factors for the Si/B^[*p*]^ intermixing (PDF)
