# Supplementary material for: Identifying and Quantifying Borate Environments in Borosilicate Glasses: 11B NMR-Peak Assignments Assisted by Double-Quantum Experiments
Source: J Phys Chem B. 2024 Dec 10;128(50):12651–67. doi: 10.1021/acs.jpcb.4c06721 (PMC11664598; doi:10.1021/acs.jpcb.4c06721)
Supplement: Supplementary file 1 — jp4c06721_si_001.pdf [file jp4c06721_si_001.pdf]

Supporting Information for

Identifying and Quantifying Borate Environments in Borosilicate Glasses: ^{11}B NMR-Peak Assignments Assisted by Double-Quantum Experiments

Baltzar Stevensson,[1] Peng Lv,[1,2] and Mattias Edén[1,*]

[1]Physical Chemistry Division, Department of Materials and Environmental Chemistry, Arrhenius Laboratory, Stockholm University, SE-106 91 Stockholm, Sweden
[2]MOE Frontiers Science Center for Rare Isotopes, Lanzhou University, Lanzhou, 730000, PR China

[*]Corresponding author. E-mail: *mattias.eden@mmk.su.se*

Contents

1. **Table S1**. Cation Field Strengths.
2. **Table S2**. Best-Fit NMR Parameters Before and After 2QF.
3. **Table S3**. Modeled Preference Factors and Average Numbers of $B^{[4]}$–O–F Linkages.
4. **Figure S1**. Single-Pulse and 2QF ^{11}B MAS NMR spectra.
5. **References**.

Table S1. Cation Field Strengths[a]

Cation Species	Cation Field Strength ($Å^{-2}$)
K^+	0.132
Rb^+/Na^+	0.147
K^+/Na^+	0.153
Na^+	0.175
Li^+/Na^+	0.197
Ba^{2+}/Na^+	0.223
Ca^{2+}/Na^+	0.265
Mg^{2+}/Na^+	0.316

[a] Cation field strength, CFS_M according to eq 4 of the as-indicated M^+/M^{2+} species of the ternary M_2O–B_2O_3–SiO_2 glasses, along with the *average* CFS, $\overline{\mathrm{CFS}} = (\mathrm{CFS}_M + \mathrm{CFS}_{\mathrm{Na}})/2$ of the M^{z+}/Na^+ cation pairs of the quaternary $[0.5M^{z+}{:}0.5Na^+]$–B_2O_3–SiO_2 glasses (see Table **1**). The cations (cation pairs) are listed according to increasing CFS ($\overline{\mathrm{CFS}}$) from top to bottom.

Table S2. Best-Fit NMR Parameters Before and After 2QF[a]

Glass	$x_B^{[4]}$[fit] [b] 1pls(2QF)	$^{11}B^{[3]}$ sites: ring (R) $\delta_{iso}^{[3]}$ (ppm)	Population	$C_{Q\eta}^{[3]}$ (MHz)	$C_Q^{[3]}$ (MHz)	$\eta_Q^{[3]}$	$W_{iso}^{[3]}$ (ppm)	non-ring (NR) $\delta_{iso}^{[3]}$ (ppm)	Population	$C_{Q\eta}^{[3]}$ (MHz)	$C_Q^{[3]}$ (MHz)	$\eta_Q^{[3]}$	$W_{iso}^{[3]}$ (ppm)
K2.0	0.660(0.463)	17.95(18.54)	0.265(0.517)	2.64(2.67)	2.58(2.62)	0.364(0.330)	2.05(2.70)	14.97(17.00)	0.075(0.020)	2.68(2.58)	2.62(2.53)	0.351(0.340)	2.28(2.70)
RbNa2.0	0.634(0.435)	17.82(18.26)	0.300(0.533)	2.64(2.66)	2.58(2.62)	0.364(0.307)	2.22(2.70)	14.56(15.67)	0.066(0.031)	2.68(2.85)	2.62(2.80)	0.351(0.340)	2.00(2.70)
Na2.0	0.635(0.472)	17.88(17.90)	0.276(0.500)	2.64(2.60)	2.58(2.57)	0.361(0.250)	2.12(2.80)	14.59(14.62)	0.089(0.028)	2.65(2.68)	2.57(2.62)	0.434(0.360)	2.28(2.80)
LiNa2.0	0.597(0.435)	17.82(18.10)	0.302(0.529)	2.66(2.67)	2.62(2.62)	0.327(0.340)	2.01(2.60)	14.95(16.10)	0.101(0.036)	2.68(2.80)	2.62(2.75)	0.351(0.326)	2.42(2.60)
MgNa2.0	0.388(0.277)	17.64(18.09)	0.409(0.592)	2.67(2.74)	2.62(2.68)	0.330(0.370)	2.38(2.53)	14.44(15.54)	0.203(0.131)	2.71(2.87)	2.66(2.82)	0.341(0.326)	2.67(3.00)
K4.0	0.713(0.423)	17.50(17.90)	0.178(0.475)	2.68(2.68)	2.63(2.63)	0.337(0.337)	2.63(2.65)	14.35(14.86)	0.109(0.102)	2.76(2.80)	2.70(2.74)	0.357(0.380)	2.77(2.65)
RbNa4.0	0.686(0.402)	17.41(18.44)	0.206(0.509)	2.63(2.76)	2.58(2.70)	0.346(0.355)	2.41(2.50)	14.06(16.00)	0.108(0.089)	2.65(2.70)	2.59(2.67)	0.350(0.292)	2.41(2.50)
Na4.0	0.650(0.414)	17.46(18.15)	0.214(0.525)	2.64(2.75)	2.59(2.68)	0.367(0.385)	2.39(2.50)	14.13(15.88)	0.135(0.061)	2.71(2.89)	2.64(2.80)	0.422(0.450)	2.39(2.50)
MgNa4.0	0.338(0.247)	17.52(18.30)	0.360(0.629)	2.73(2.84)	2.67(2.77)	0.367(0.400)	2.65(2.67)	13.83(14.54)	0.303(0.124)	2.74(2.81)	2.68(2.76)	0.354(0.314)	2.65(2.67)
σ [c]	0.007(0.014)	0.15(0.20)	0.020(0.030)	0.03(0.06)	0.03(0.06)	0.030(0.060)	0.15(0.30)	0.40(0.50)	0.020(0.030)	0.03(0.06)	0.03(0.06)	0.030(0.060)	0.15(0.30)

Glass	$x_B^{[4]}$[fit] [b] 1pls(2QF)	$^{11}B^{[4]}$ sites: 2Si $\delta_{iso}^{[4]}$ (ppm)	Population	$C_{Q\eta}^{[4]}$ [d] (MHz)	$W_{iso}^{[4]}$ (ppm)	3Si $\delta_{iso}^{[4]}$ (ppm)	Population	$C_{Q\eta}^{[4]}$ [d] (MHz)	$W_{iso}^{[4]}$ (ppm)	4Si $\delta_{iso}^{[4]}$ (ppm)	Population	$C_{Q\eta}^{[4]}$ [d] (MHz)	$W_{iso}^{[4]}$ (ppm)
K2.0	0.660(0.463)	1.00(0.97)	0.157(0.199)	0.37(0.37)	2.59(3.04)	−0.37(−0.21)	0.299(0.175)	0.31(0.31)	1.90(2.17)	−1.90(−1.90)	0.204(0.089)	0.24(0.24)	1.92(2.61)
RbNa2.0	0.634(0.435)	1.00(1.20)	0.143(0.223)	0.37(0.37)	2.79(3.00)	−0.32(−0.08)	0.315(0.154)	0.31(0.31)	1.96(1.97)	−1.90(−1.70)	0.175(0.058)	0.24(0.24)	1.86(2.01)
Na2.0	0.635(0.472)	1.00(1.10)	0.121(0.154)	0.37(0.37)	2.85(3.00)	−0.12(−0.06)	0.284(0.217)	0.31(0.31)	1.87(2.07)	−1.68(−1.70)	0.231(0.101)	0.24(0.24)	1.96(2.00)
LiNa2.0	0.597(0.435)	1.05(1.50)	0.123(0.149)	0.37(0.37)	2.95(3.00)	−0.04(0.02)	0.291(0.222)	0.31(0.31)	1.96(2.37)	−1.63(−1.74)	0.183(0.065)	0.24(0.24)	1.95(3.00)
MgNa2.0	0.388(0.277)	1.05(1.30)	0.077(0.089)	0.37(0.37)	2.60(3.00)	0.03(0.10)	0.193(0.134)	0.31(0.31)	1.77(2.13)	−1.62(−1.62)	0.118(0.053)	0.24(0.24)	2.04(2.31)
K4.0	0.713(0.423)	1.00(1.30)	0.057(0.099)	0.37(0.37)	2.30(3.00)	−0.58(−0.28)	0.254(0.239)	0.31(0.31)	1.77(2.28)	−2.13(−1.96)	0.402(0.086)	0.24(0.24)	1.88(2.02)
RbNa4.0	0.686(0.402)	1.30(1.40)	0.040(0.073)	0.37(0.37)	2.07(2.35)	−0.48(−0.22)	0.289(0.240)	0.31(0.31)	1.85(2.14)	−2.10(−1.90)	0.358(0.090)	0.24(0.24)	1.83(2.06)
Na4.0	0.650(0.414)	1.07(1.30)	0.050(0.090)	0.37(0.37)	2.60(2.60)	−0.40(−0.26)	0.283(0.237)	0.31(0.31)	2.00(2.17)	−2.02(−2.02)	0.318(0.086)	0.24(0.24)	1.92(1.92)
MgNa4.0	0.338(0.247)	1.13(1.37)	0.042(0.048)	0.37(0.37)	2.60(2.40)	−0.17(−0.03)	0.182(0.138)	0.31(0.31)	1.96(2.12)	−1.96(−1.80)	0.113(0.061)	0.24(0.24)	1.94(2.20)
σ [c]	0.007(0.014)	0.17(0.35)	0.010(0.031)	n.d.(n.d.)	0.09(0.17)	0.17(0.35)	0.016(0.031)	n.d.(n.d.)	0.09(0.17)	0.17(0.35)	0.016(0.031)	n.d.(n.d.)	0.09(0.17)

[a] Best-fit parameters obtained by deconvoluting the single-pulse ^{11}B MAS NMR spectra and each 1Q projection of the 2Q–1Q correlation 2D NMR spectra (2QF; data in parentheses). The data are grouped according to the sets of $\{B^{[4]}(m\mathrm{Si})\}$ and $\{B^{[3]}(\mathrm{R}), B^{[3]}(\mathrm{NR})\}$ sites, where R and NR represents trigonal ^{11}B sites in "ring" and "non-ring" motifs, respectively. The NMR-parameter notation is introduced in section **2.4**.

[b] Net fractional population of the best-fit $\{x_B^{[4]}(m\mathrm{Si})\}$ set: $x_B^{[4]}[\mathrm{fit}] = x_B^{[4]}(2\mathrm{Si}) + x_B^{[4]}(3\mathrm{Si}) + x_B^{[4]}(4\mathrm{Si})$. The minor discrepancy to its $x_B^{[4]}$ counterpart ("1pls") listed in Table **1** stems partially from uncertainties in the spectral deconvolutions but primarily from the centerband ST $^{11}B^{[4]}$ resonance intensity, which was not accounted for by the spectral deconvolutions and leading to an overestimation of $x_B^{[4]}$ by ≈ 0.03.

[c] The uncertainties of the best-fit data are $\pm 1\sigma$ with σ given for each entity.

[d] This parameter was kept constant at the as-specified value and data uncertainties were not determined (n.d.).

Table S3. Modeled Preference Factors and Average Numbers of $B^{[4]}$–O–F Linkages[a]

Glass	fraction			$P(B^{[4]}–O–F)$			$\overline{N}(B^{[4]}–O–F)$		
	x_{Si}	$x_B x_B^{[3]}$	$x_B x_B^{[4]}$	Si	$B^{[3]}$	$B^{[4]}$	Si	$B^{[3]}$	$B^{[4]}$
Na2.0	0.500	0.227	0.273	1.16	1.38	0.50	2.50	0.94	0.56
MgNa2.0	0.500	0.323	0.177	1.03	1.27	0.54	2.36	1.23	0.41
Na4.0	0.667	0.158	0.176	1.09	1.38	0.40	3.06	0.66	0.28
MgNa4.0	0.667	0.233	0.100	0.99	1.33	0.49	2.89	0.93	0.18

[a] Molar fractions of Si, $B^{[3]}$, and $B^{[4]}$ (Table **3**) and preference factors $P(B^{[4]}–O–F)$ for forming $B^{[4]}$–O–F linkages with F = {Si, $B^{[3]}$, $B^{[4]}$}, calculated from the as-observed average number of $B^{[4]}$–O–F linkages in the glass models, $\overline{N}(B^{[4]}–O–F)$, relative to that predicted for an unconstrained statistical $B^{[4]}$–O–F linkage formation.[S1,S2] Note that each value of $\overline{N}(B^{[4]}–O–Si)$ is identical to that of $\overline{m}$ listed in Table **3**. The preference factor $P(B^{[4]}–O–F)$ is unity for statistically/randomly distributed F={Si, $B^{[3]}$, $B^{[4]}$} species in the second coordination sphere of $B^{[4]}$, while larger (lower) values mark a *preference* and *reluctance* of $B^{[4]}$–O–F linkage formation, respectively.[S1–S3]

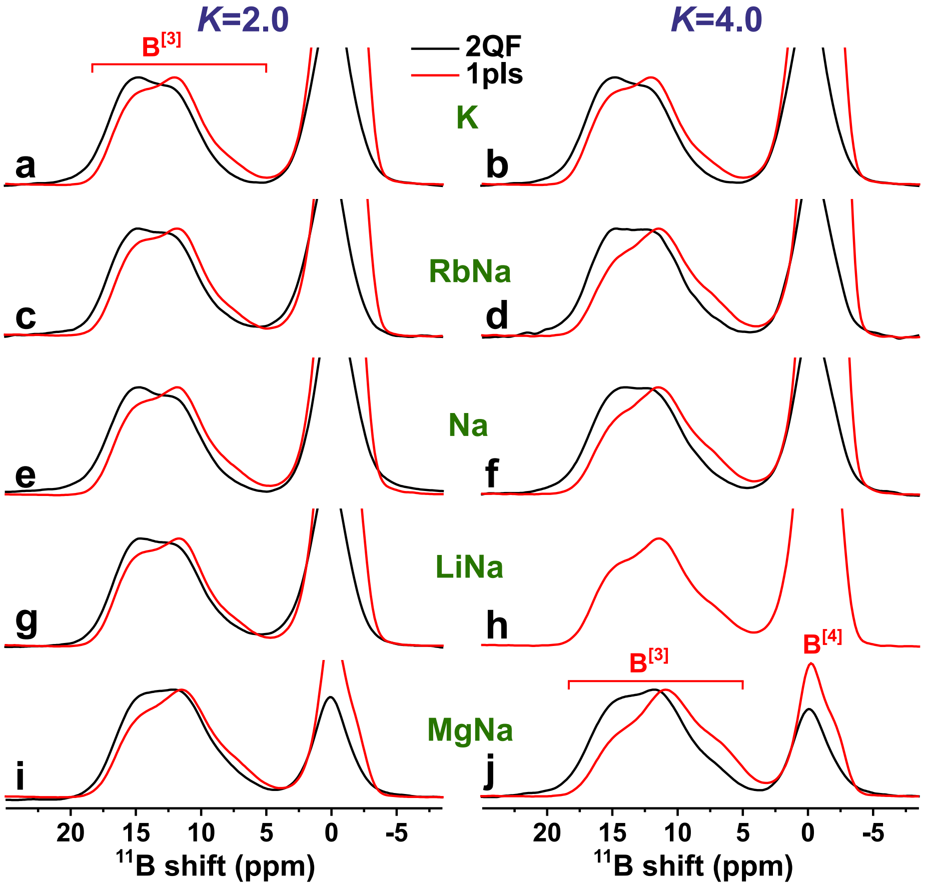

Fig. S1. The ^{11}B NMR spectra of Fig. **3** normalized to equal maximum $^{11}B^{[3]}$ peak-intensity of the single-pulse and 2QF spectra.

References

(S1) Lv, P.; Stevensson, B.; Yu, Y.; Wang, T.; Edén, M. BO_3/BO_4 Intermixing in Borosilicate Glass Networks Probed by Double-Quantum ^{11}B NMR: What Factors Govern BO_4–BO_4 Formation?. *J. Phys. Chem. C* **2023**, *127*, 20026–20040.

(S2) Lv, P.; Stevensson, B.; Mathew, R.; Wang, T.; Edén, M. Sub-Nanometer-Range Structural Effects From Mg^{2+} Incorporation in Na-Based Borosilicate Glasses Revealed by Heteronuclear NMR and MD Simulations. *J. Phys. Chem. B* **2024**, *128*, 6922–6939.

(S3) Yu, Y.; Stevensson, B.; Edén, M. Direct Experimental Evidence for Abundant BO_4–BO_4 Motifs in Borosilicate Glasses from Double-Quantum ^{11}B NMR Spectroscopy. *J. Phys. Chem. Lett.* **2018**, *9*, 6372–6376.
